# Classification, Synthesis, and Application of Luminescent Silica Nanoparticles: a Review

**DOI:** 10.1186/s11671-019-3006-y

**Published:** 2019-06-04

**Authors:** Lei Li, Wei Wang, Jianguo Tang, Yao Wang, Jixian Liu, Linjun Huang, Yanxin Wang, Fengxiang Guo, Jiuxing Wang, Wenfei Shen, Laurence A. Belfiore

**Affiliations:** 1 Institute of Hybrid Materials, National Center of International Research for Hybrid Materials Technology, National Base of International Science & Technology Cooperation, College of Materials Science and Engineering, Qingdao University, 266071 Qingdao, People’s Republic of China; 2 Institute of Oceanographic Instrumentation, Shandong Provincial Key Laboratory of Marine Monitoring Instrument Equipment Technology, National Engineering and Technological Research Center of Marine Monitoring Equipment, Qilu University of Technology (Shandong Academy of Sciences), Qingdao, 266001 China; 3 Department of Chemical and Biological Engineering, Colorado State University, Fort Collins, CO 80523 USA

**Keywords:** Luminescent silica nanoparticles, Classification, Synthesis, Application

## Abstract

Luminescent materials are of worldwide interest because of their unique optical properties. Silica, which is transparent to light, is an ideal matrix for luminescent materials. Luminescent silica nanoparticles (LSNs) have broad applications because of their enhanced chemical and thermal stability. Silica spheres of various sizes could be synthesized by different methods to satisfy specific requirements. Diverse luminescent dyes have potential for different applications. Subject to many factors such as quenchers, their performance was not quite satisfying. This review thus discusses the development of LSNs including their classification, synthesis, and application. It is the highlight that how silica improves the properties of luminescent dye and what role silica plays in the system. Further, their applications in biology, display, and sensors are also described.

## Introduction

Luminescent materials are widely applied because of their special optical properties [1]. However, their application is limited by many restrictions, such as low hydrophobicity and biocompatibility, or owing to disadvantage, such as high toxicity, poor biocompatibility, and low absorbance [2–5]. Thus, it is necessary to modify luminescent materials to satisfy the requirements of practical applications.

LSNs with improved properties have attracted more and more attention in biology [6, 7], lighting [8], and sensors [9]. Their characteristic optical properties make them unique in optical materials [10]. Silica is transparent to light which makes silica an ideal candidate as matrix for fluorescent materials. Thermodynamic and chemical stability are also important factors as a matrix and silica coincides with these basic factors [11]. Moreover, the surface of silica can be easily modified, allowing further functionalization with various functional groups to adapt diverse requirements [12]. Silica having many of the above advantages is naturally an ideal substrate for improving the properties of luminescent materials [13]. Multifunctional nanosystems can be created by assembling, encapsulating, or integrating one or more different nanomaterials within and on the surface of silica nanoparticles using different processes [11]. As modifying luminescent materials, LSNs with the excellent properties are attracting more and more attention in frontier researches [14]. Montalti et al. summed up many excellent researches in medical imaging with organic dyes doped silica [6]. Silica provides a stable and multifunctional platform for phosphors, but long-term toxicity needs to be studied. Michael Schäfrling demonstrated the art of luminescent sensors [9]. Selectivity and sensitivity are the core of the sensor materials. Zou Hua et al. elaborated on the means of organic silica modification. Nanocomposites have superior properties to separate components [15]. There are many wonder reviews focusing on a specific area such as biology [6, 7, 16], but lacking a systematic introduction to LSNs and their excellent performance in other fields.

This review starts with the classification of LSNs following with their synthetic methods. The categories of LSNs are systematically established based on the classification of luminescent materials. In terms of chemical properties and luminescence mechanisms, organic molecule dyes, luminescent metal, and quantum dots (QDs) doped phosphors are three typical phosphors, all of which have their own unique luminescence mechanisms and advantages as representatives of LSNs [17–19]. The highlight is how the silica enhances the properties of phosphors. With the deficiency of luminescent materials applications, the possible strategies are discussed to improve their performances for LSNs. It involves not only biological applications but also displays and sensors.

## Classifications of LSNs

Luminescence emitting various brightness has great value in material fields [20]. A lot of researches on the modification of luminescent materials have been carried out around how to improve the signal-to-noise ratio, stability, and environmental adaptability for the potential applications. The introduction of antenna ligands in lanthanide complexes to enhance luminescence performance is a typical example of modification. Silica is a good matrix for mixing materials with different functions and different chemical properties. Phosphors have been doped into silica matrix to modify their natural defects and improve their properties, which is advantageous for broad applications with facile modified and non-toxic silica surfaces and protection for luminescent dyes. With multifunction and tunable adaptability, LSNs have attracted more and more attention. Among all the luminescent phosphors, organic luminescent molecules, luminescent metal-doped phosphors and QDs are the three most representative categories which deserve highlighting. So the above three dyes are shown as typical LSNs in combination with silica. Representative examples are listed in Table 1.

**Table 1:** The composition and synthetic methods of reported LSNs

Materials		Synthetic methods	Ref
Organic luminescent molecules doped LSNs	FITC-APS@silica	Stöber method	[21]
	TRITC:SiO_2_@FITC:SiO2	Modified Stöber method	[22]
	Aminoccyanine dye-silica hybrid nanopartcles	Reverse microemulsion	[23]
	AIE-F127-SiO_2_	Sol-gel method	[24]
	HPTS-adsorbed Ag@SiO_2_	Stöber method	[25]
	Rhodamine-conjugated silica	Direct micelles assistant method	[26]
	An18-SiO_2_	Stöber method	[27]
	Y_2_O_3_:Eu^3+^@SiO_2_ with FITC	Stöber method	[28]
	FSCHP	Stöber method	[29]
Luminescent metal-doped LSNs	Eu@Si-OH	Reverse microemulsion	[30]
	Eu@Si-NH_2_		
	NaGdF_4_:Yb,Er@SiO_2_@Eu (TTA)3Phen	Reverse microemulsion	[31]
	Eu-mesoporous silica nanospheres	Modified sol-gel method	[32]
	SiO_2_-[Eu (TTA)_3_(Bpy-Si)]	Modified Stöber method	[33]
	Perovskite QD/silica composites	Other method	[34]
	Mesoporous silica particles integrated with all-inorganic CsPbBr3 absorption		[35]
	CD/silica composites heating silica film		[36]
	Silica@EuCP	Solvothermal method	[37]
	Ru (bpy)_3_ doped silica	Reverse microemulsion	[38]
	Ru (bpy)_3_@SiO_2_:Gd	Reverse microemulsion	[39]
	Ru (bpy)_3_@SiO_2_	Stöber method	[40]
	Er,Yb:GdVO_4_@SiO_2_	Stöber method	[41]
QDs-doped LSNs	Carbon dot-silica-phosphor composite	Stöber method	[42]
	CdSe/CdS/ZnS@SiO_2_	Stöber method	[43]
	BAM-SiO_2_-CdSe MQDs	Reverse microemulsion	[44]
	Quantum dot/SiO_2_/Au	Reverse microemulsion	[45]
QDs-doped LSNs	CdS/CdSe/CdS@SiO_2_	Reverse microemulsion	[46]
	FL-SiO_2_ (carbon dots)	Direct micelles assistant method and calcination	[47]
	SiO_2_/Fe_2_O_3_-CdSe	Reverse microemulsion	[48]
	Silica encapsulated polymer dots	Stöber method	[49]
Silica quantum dots	Organosilica nanocrystals	Stöber method	[50]
	Silica NPs	Hydrothermal method	[51]

### Organic Luminescent Molecules Doped LSNs

**Fig. 1:**
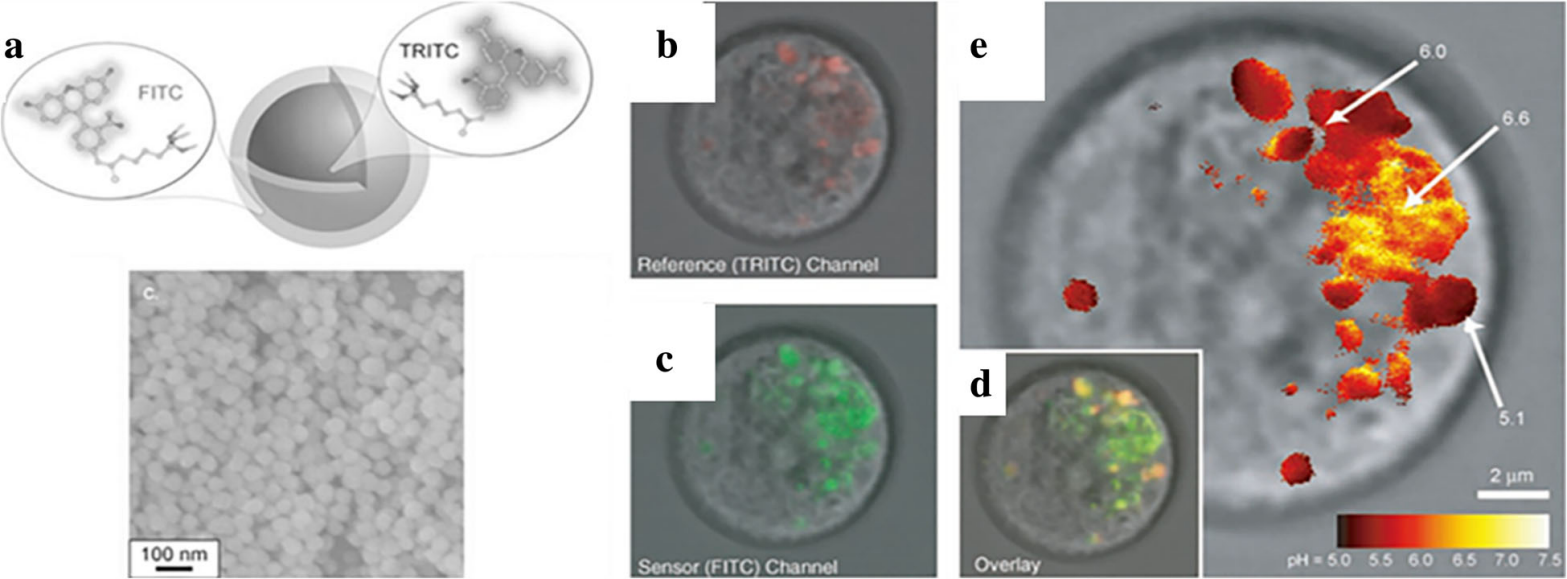
**a** The formation diagram and scanning electron microscope (SEM) image of dual-emission fluorescent silica nanoparticles with reference dye (TRITC) and sensor dye (FITC); the confocal fluorescence microscopy image of RBL mast cells (red as TRITC silica particles and green as AlexaFluor 488-Cholera toxin B); confocal fluorescence microscopy images of RBL mast cells as pH sensors. **b** For reference channel, **c** for sensor channel, **d** for the overlaid images, and **e** false-color ratiometric imaging for pH values calculated according to the experiments [22]

Organic luminescent molecules are important luminescent materials with π-conjugated ring structures and small size [16]. However, non-specific labeling and bleaching hinder their application. Organic dyes doped silica nanoparticles are widely studied with excellent stability, selectivity and biocompatibility [52, 53]. Van Blaaderen et al. [21] made a preliminary attempt to synthesize luminescent silica spheres. Fluorescein isothiocyanate (FITC) was coated on the surface of silica with the help of APS ((3-aminopropyl)triethoxysilane) which provided a feasible way to combine dyes with silica by covalent bonds. Inspired by this process, Andrew et al. [22] synthesized dual-emission fluorescent silica nanoparticles with two layers. Two dyes, tetramethylrhodamine isothiocyanate (TRITC) and FITC, were conjugated to the silica by means of APS in an anhydrous nitrogen environment. The schematic diagram and SEM image (Fig. 1a) showed the nanostructures of the nanoparticles. Silica with TRITC was synthesized firstly as the core of the dual-emission nanoparticles and FITC was conjugated on the surface of the core with further tetraethoxysilane (TEOS). The synthesized dual emission fluorescent silica nanoparticles investigated the intracellular pH value successfully in rat basophilic leukemia mast cells (RBL-2H3) in Fig. 1b–d.

**Fig. 2:**
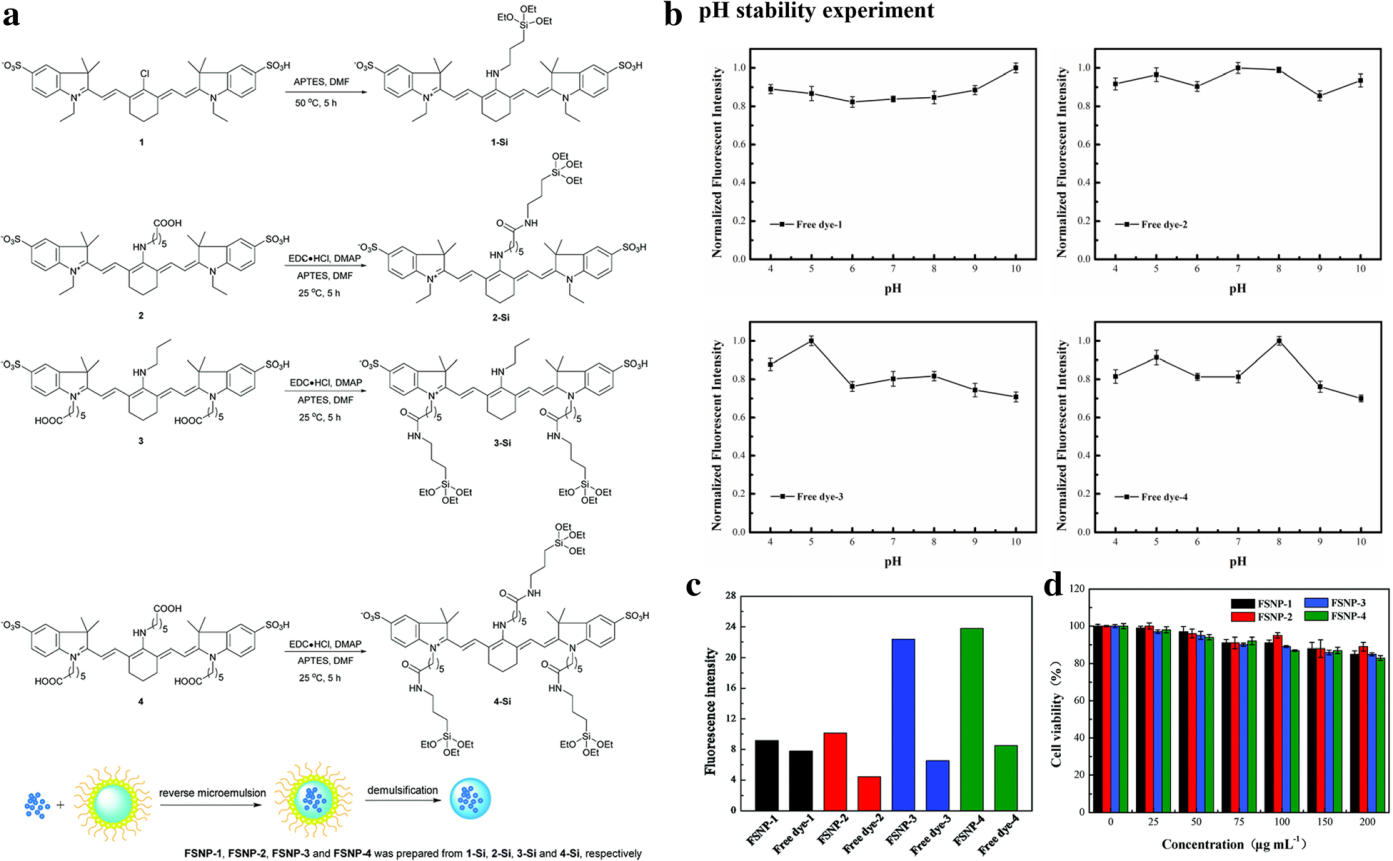
**a** The mechanism of FSNP-1, FSNP-2, FSNP-3, and FSNP-4. **b** The normalized intensity in different pH values of all the samples. **c** Emission intensity of the FSNPs and free dyes. **d** Showing after incubated with FSNPs for 24 h, the viability of the raw264.7 macrophage cells [23]

In order to improve the photostability of organic fluorophores, silica encapsulation is a commonly used method of modification. Long Jiao et al. [23] chose four aminocyanine dyes as the near-infrared (NIR) fluorescent probes and coupled them correspondingly with 3-aminopropyltriethoxysilane (APTES), a common silane coupling agent. TEOS hydrolyzed with the treated NIR dyes in the microemulsion system. The cyanine-loaded fluorescent silica nanoparticles (FSNPs) were obtained after centrifugation and washing. The whole process was shown in Fig. 2a. As it can be seen in Fig. 2b, the four FSNPs encapsulated into the silica showed better fluorescence intensity pH stability than that of free dyes. The four FSNPs improved their brightness at the same time (Fig. 2c). They tested their photostability in living cells by a confocal laser scanning microscope (CLSM). FSNP-3 and FSNP-4 (more anchoring sites) got improved photostability than that of free dyes while FSNP-1 and FSNP-2 did not get any improvement. More anchoring sites reinforced the structure of the dye molecule. A dye with rigid structure had a less nonradiative decay and hard intramolecular rotation which make the dye brighter. Silica layer can protect the encapsulated materials which has reinforced molecule structure and improve their brightness without photobleaching. FSNP-3 and 4 also had low biological toxicity according to the methyl tetrazolium (MTT) method in Fig. 2d. Biocompatibility is another advantage of silica.

**Fig. 3:**
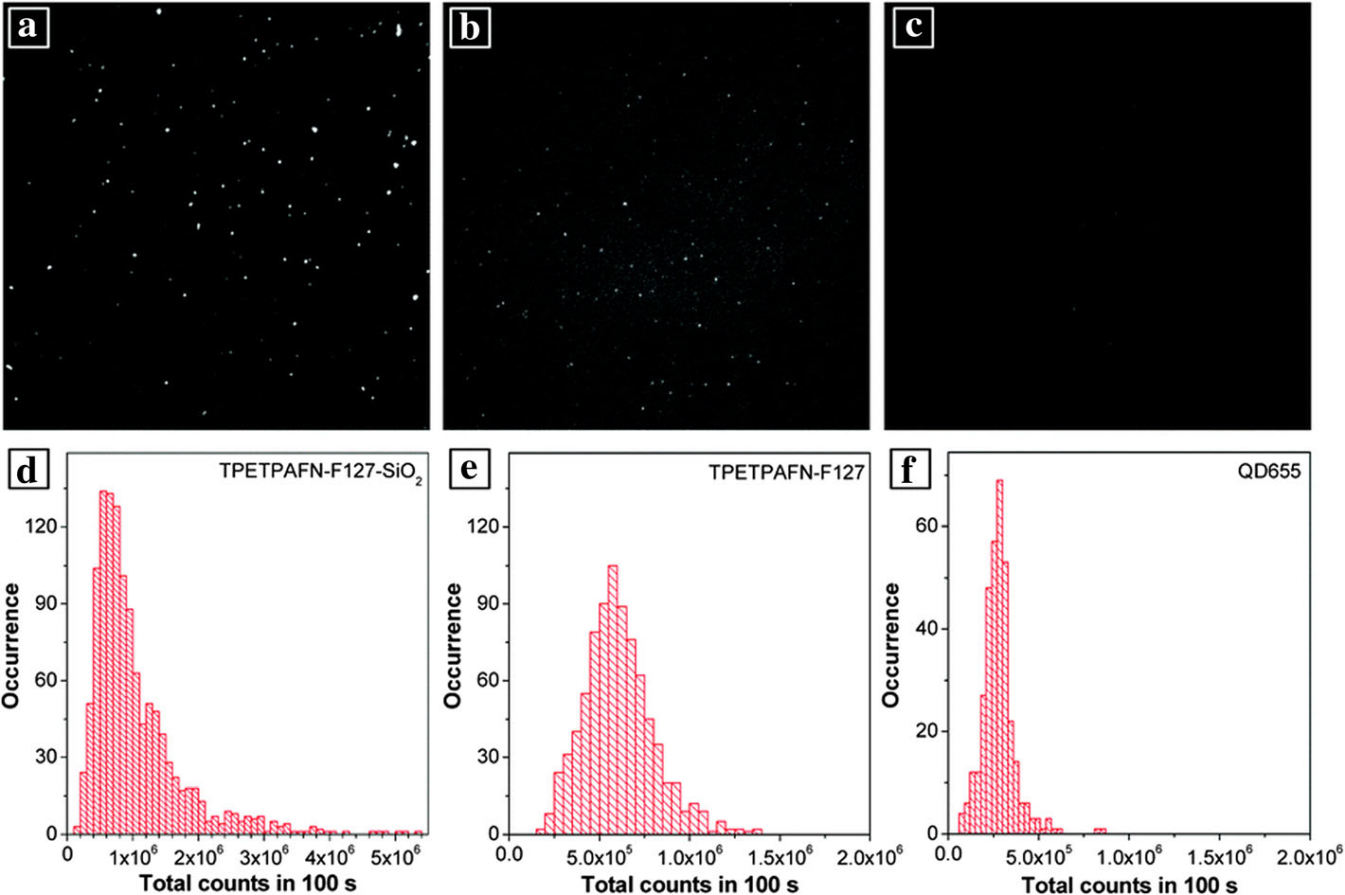
**a**, **d** Showing the images of fluorescence and the histograms of photons of TPETPAFN-F127-SiO_2_ NPs, corresponding **b**, **e** for TPETOAFN NPs and **c**, **f** for commercial QD655 [24]

Agglomeration is one of the main reasons of quenching for most luminescent dyes. Phosphors can keep in an appropriate concentration with silica steadily. Aggregation-induced emission luminogens (AIEgens), unlike the traditional luminophores, do not suffer from this issue. Conversely, aggregation leads to strong emission [54]. To improve the performance of AIEgens in biological fields, many polymer matrices are used to encapsulate AIEgens. Moreover, there are some other issues that can cause AIEgens quenching, such as water and oxygen, which has a negative effect on application. Silica can prevent them from the quenchers [55]. Based on these analysis above, TPETPAFN (a typical fluorogen consisting of two tetraphenylethylene pendants and an intramolecular charge transfer core), an AIEgen, was biofunctionalized by F127 (poly (ethylene glycol)-block-poly (propylene glycol)-block-poly (ethylene glycol)) to form the core micelle [24]. TEOS was hydrolyzed to obtain silica shell coated on the core micelle via modified sol-gel method. As Fig. 3 showed, the synthetic TPETPAFN-F127-SiO_2_ nanoparticles exhibited better photoluminescence properties benefited from the protection of the silica shell.

### Luminescent Metal-Doped LSNs

**Fig. 4:**
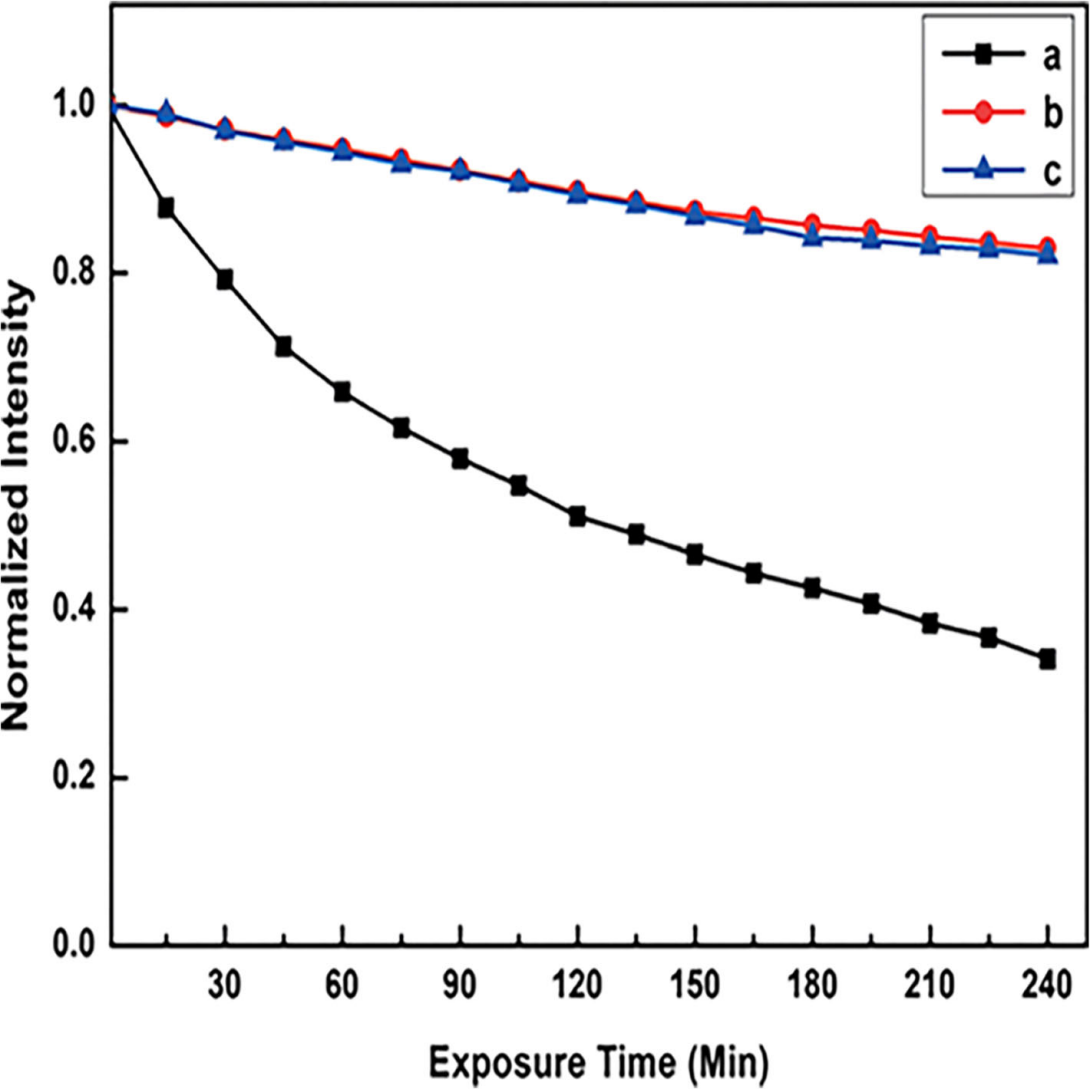
The curves of fluorescence intensity changing with exposure time under 365 nm irradiation, **a** parent Eu complex in CHCl_3_ solution, **b** Eu@Si-NH_2_, and **c** Eu@Si-OH nanoparticles in phosphate-buffered saline (PBS) buffer solution [30]

Rare-earth metal [56] and transition metal [57] are common luminescent metal materials based on the charge transfer transition. Luminescence after complexing with the ligand is the most obvious feature of this material. There are two main mechanisms of metal luminescence, LMCT (ligand to metal charge transition) and MLCT (metal to ligand charge transition). Lanthanide metals and transition metals are their typical examples, respectively. Due to abundant electronic energy levels, there are diverse luminescent metals that have great potential for application in the field of luminescence with different emission [58]. Noble metals with LSPR have been widely used in enhanced luminescence materials and are involved in this section. Nevertheless, low sensitization efficiency and quenching limit the applications of luminescent metals [59]. In order to improve their photostability and biocompatibility, Francis et al. added a substituted silyl group into the ligands for the further modifications [30]. Eu@Si-OH nanoparticles were obtained after coating silyl group modified Eu complexes with silica via reverse microemulsion method. The product finally got amine functionalized with APTES as Eu@Si-NH_2_ nanoparticles. The silica layer kept the complexes from the quenchers (OH and NH_2_ groups). As a result, both of them showed better photostability in Fig. 4. Eu@Si-NH_2_ nanoparticles exhibited good performances in bioimaging.

Ezquerro et al. incorporated Ir complexes, MLCT luminescent complex, into the silica framework for enhanced stability and photophysical properties via sol-gel process [60]. With the protection of silica, these phosphors showed excellent stability under not only ambient conditions but also harsh environments which have further application in white light-emitting diode (WLED).

**Fig. 5:**
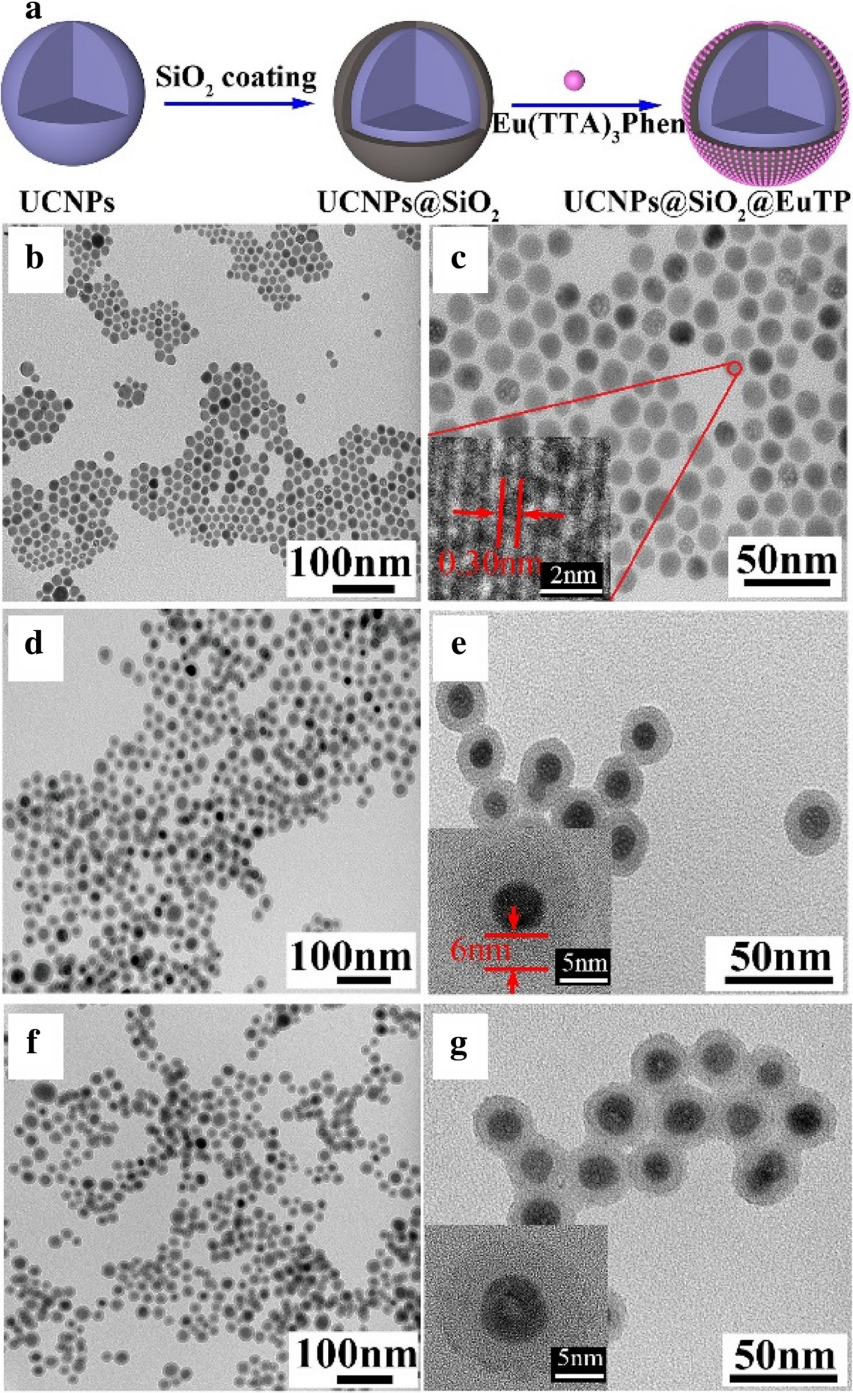
**a** The formation of UCNPs@SiO_2_@EuTP and TEM (transmission electron microscope) images of the samples; **b**, **c** for UCNPs, **d**, **e** for UCNPs@SiO_2_, and **f**, **g** for UCNPs@SiO_2_@EuTP [31]

Y. Li et al. [31] synthesized oleic acid stabilized upconversion nanoparticles (UCNPs). Then they coated a silica layer on the UCNPs via a microemulsion method and water-soluble UCNPs were obtained. Introducing Eu (TTA)_3_phen complexes into the system, they synthesized NaGdF_4_:Yb,Er@SiO_2_@Eu (TTA)_3_Phen (UCNPs@SiO_2_@EuTP) nanospheres. The surface quenching was suppressed after silica coating and as a result the emission intensity enhanced as shown in Fig. 5. The water-soluble nanoparticles with two different emissions were obtained with the help of silica.

**Fig. 6:**
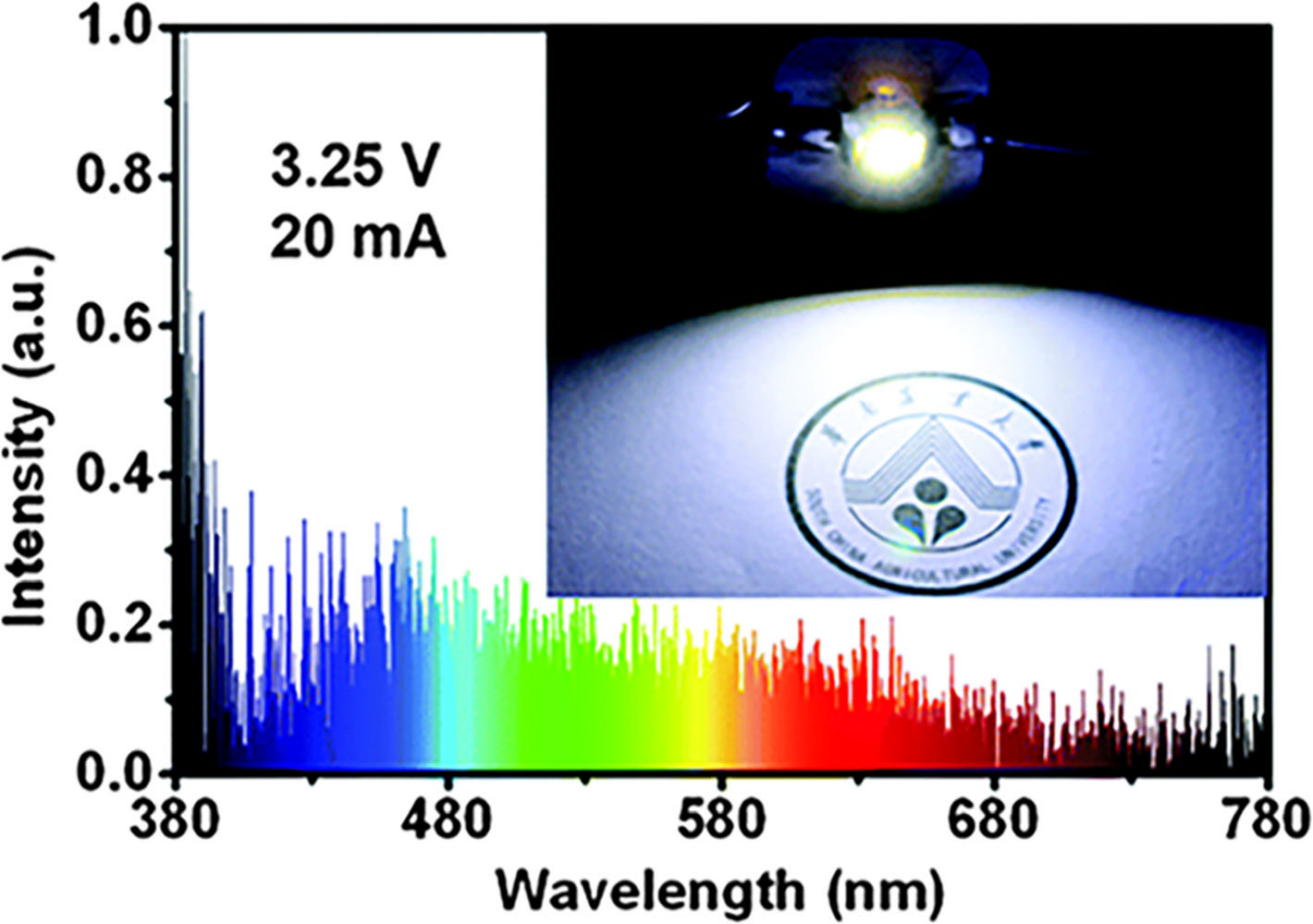
The performances of CDSP-based WLED: emission spectra and photograph [42]

Chen et al. [42] successfully used carbon dots (CDs) and rare-earth ions for WLED. They synthesized CDs by one-pot organic pyrolysis method, which had a maximum of blue emission at 470 nm, and two excitation peak at 251 and 364 nm, respectively. To get white light-emitting composite, CDs were used as the blue emission core and Sr_2_Si_5_N_8_:Eu^2+^ phosphor was used as the orange emission component. CDs were put into the Stöber system. As TEOS hydrolyzed, CDs would be coated by a silica layer with red phosphor. The carbon dot-silica-phosphor composite (CDSP) was synthesized after centrifuging, drying, and grinding. CDSP had a broad absorption ranging from the UV (ultraviolet) to yellow region (200–600 nm), especially strong in the UV region. After testing excitation at different wavelengths, they found that the CDSP got the closet Commission Internationale de l’Eclairage (CIE) coordinate (0.32, 0.32) to that of pure white light (0.33, 0.33) at the excitation at 400 nm in Fig. 6. And it was a good attempt to get the emission of CDSP by tuning the mass ratio of CD and the phosphor. Under excitation at 400 nm, they got the nearest mass ratio (3.9% (0.32, 0.32) and 5.1% (0.34, 0.32)) to white emission. CDSP showed better white emission (0.30, 0.31) in light-emitting diode (LED) packaging than that of CD&P (mixing up with CDs and phosphor directly) (0.28, 0.29). Two components dispersed homogeneously with silica and decreased the probability of aggregation and phase separation. Finally, they got a WLED with CDSP powder on a UV diode chip (375 nm), and a white light (0.30, 0.33) was obtained. The color rendering index (CRI) was about 94, higher than that of the YAG:Ce based commercial WLED (CRI < 75).

**Fig. 7:**
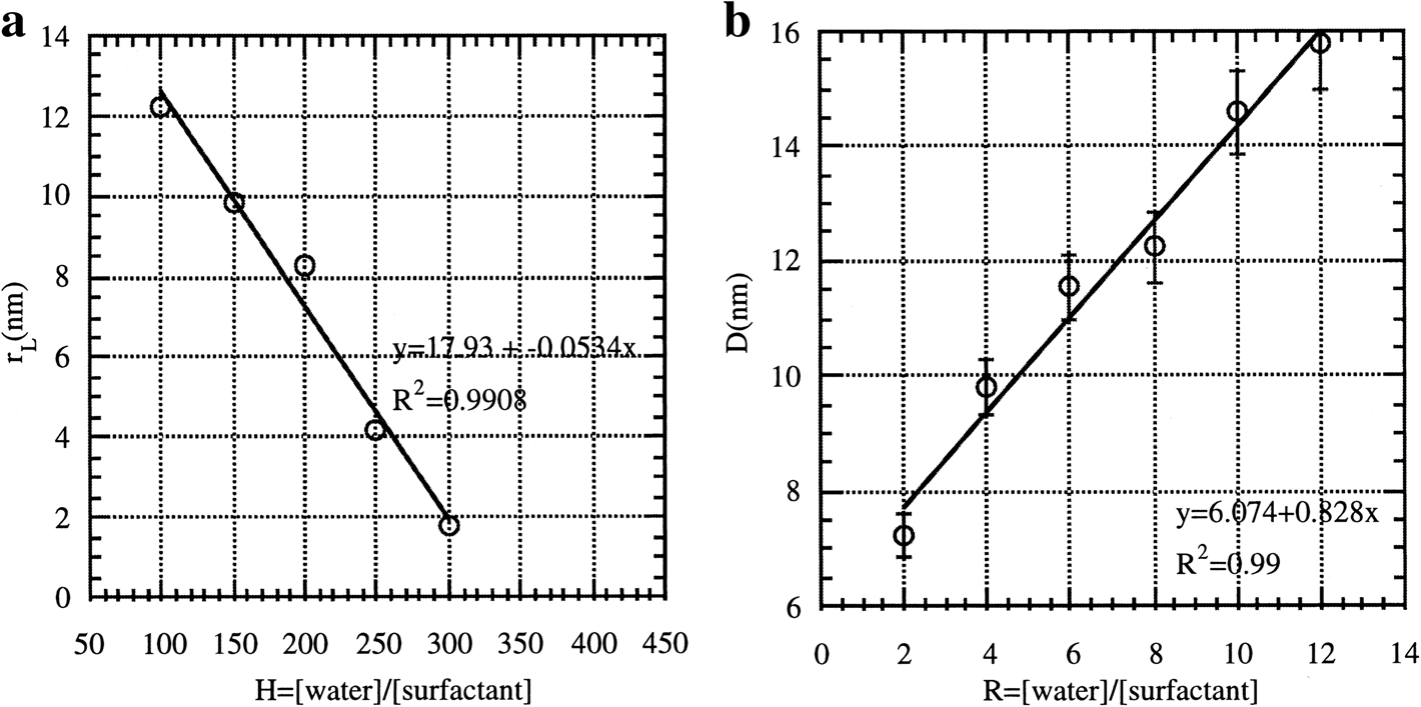
**a** The size change as a function of H (*R* = 4 and *X* = 1); **b** when *R* is variable, the size change of Ag clusters [61]

**Fig. 8:**
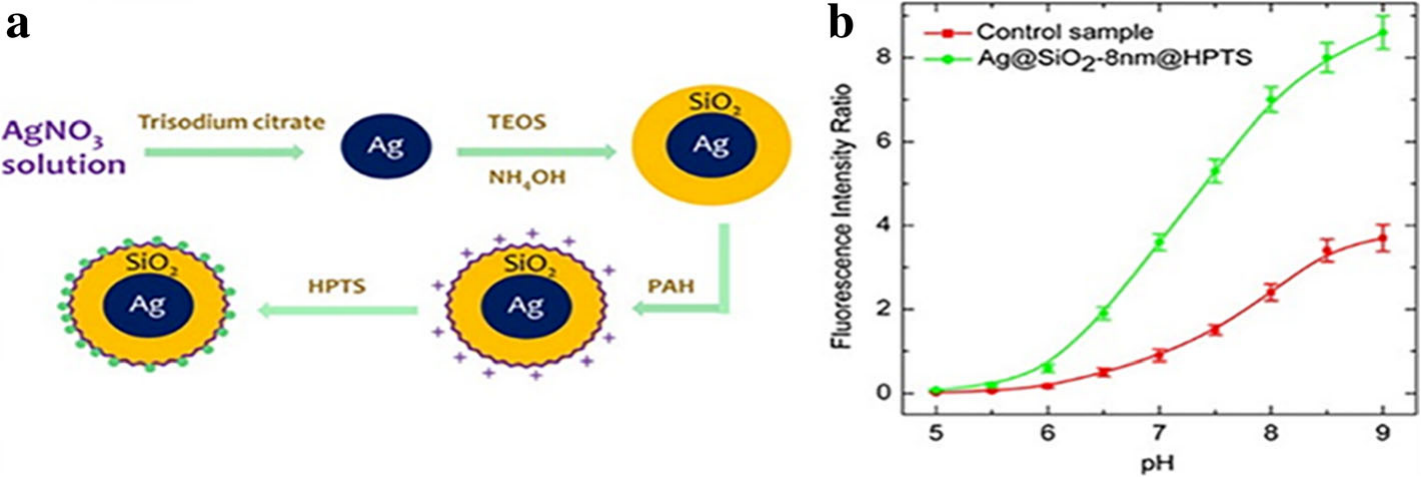
**a** The progress of synthesis of HPTS-adsorbed Ag@SiO_2_ nanoparticles. **b** The fluorescence intensity ratio of Ag@SiO_2_-8 nm@HPTS (green) and control sample (red) [25]

Silica is commonly used as protective layer for luminescent materials to keep proper distance from noble metals in order to enhance fluorescence. This is due to the standing oscillation of free electrons caused by light. In order to enhance the luminescence, it needs to be kept in an appropriate distance between dyes and noble metal particles. As for noble enhanced materials, metal nanoparticles themselves can caused the chromophores quenching (within 5 nm) but their fluorescence can enhance up 100-fold (at around 10 nm). In the early research, Tuo Li et al. [61] synthesized Ag nanoparticles with the silica shell in the microemulsion matrix (Ag/SiO_2_ nanoparticles). The reagents needed to produce silica (TEOS and cyclohexane) were injected into the microemulsion after silver reduced. They carefully studied the effects of different conditions (water/surfactant for R and water/TEOS for H) on the Ag/SiO_2_ nanoparticles and the results showed as Fig. 7. It is a good path to coat a uniform and thick silica layer on the core not only Ag but also other nanoparticles with microemulsion system. What Zhenhua Bai et al. [25] did is a good example. 8-Hydroxypyrene-1, 3, 6-tresulfonic acid (HPTS), a kind of fluorescent pH-sensitive dyes, is suitable to make as intracecellular pH sensors owing to its unique advantages. But extreme pH conditions made it insensitive. When the solution is acidic, its fluorescence efficiency would decrease significantly. HPTS-adsorbed Ag@SiO_2_ nanoparticles (Fig. 8a) were prepared based on noble metal-enhanced fluorescence effect. It could be seen from Fig. 8b that Ag@SiO_2_-8 nm@HPTS showed better fluorescence intensity especially in the extreme pH conditions.

### QDs-Doped LSNs

Because of quantum confinement effect, QDs exhibit excellent luminescence properties whether they are semiconductor QDs, carbon QDs, or other types. Recently, numerous studies have focused on the applications of QDs in optical devices. Sometimes, their properties are not good enough to adapt the complex applications. Necessary modification is imperative and silica is a suitable matrix [1].

**Fig. 9:**
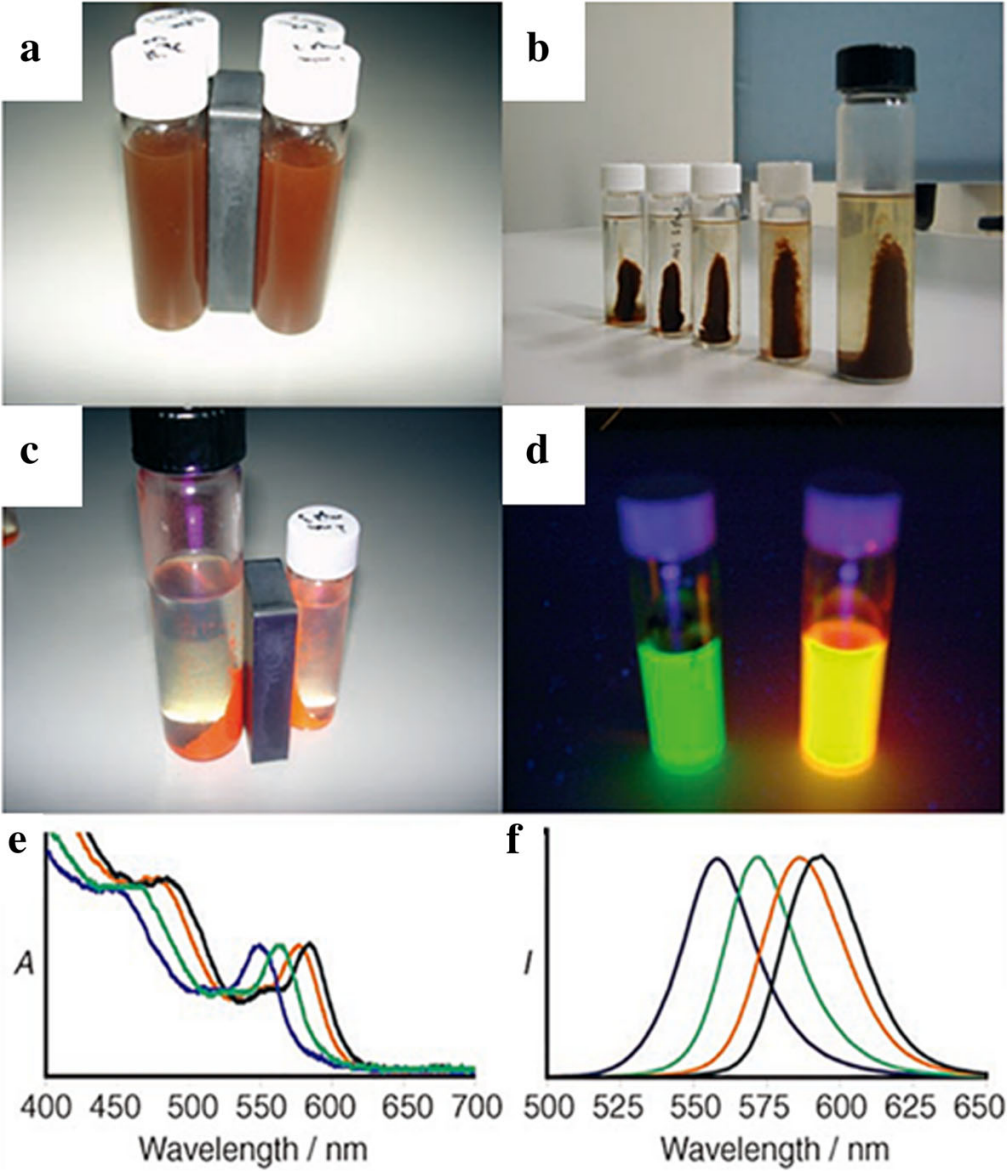
The photographs under normal light to prove the magnetic modification (**a**, **b**). **c** The photograph under UV light to prove both the magnetic and luminescence properties. **d** The UV photograph of green and orange magnetic quantum dots (MQDs). **e**, **f** The luminescence spectra of SiO_2_-MQDs in PBS solutions (**e** for absorption and **f** for emission) [44]

To realize the combination of biolabel and magnetic resonance imaging, CdSe QDs were coated on the magnetic Fe_2_O_3_ core by a silica layer with NH_2_ group. The associated pictures and characterizations were showed in Fig. 9. Combined NH_2_ group with bio-anchored membrane (BAM), 4 T1 mouse breast cancer cell membranes showed specific labeling with BAM-SiO_2_-CdSe MQDs [44]. With the biocompatibility and magnetic, the multifunctional luminescence nanoparticles would gain broad applications in medicine.

To broaden QDs application, it is necessary to modify their water solubility and non-toxicity. Silica shows great potential in QDs’ modification. Yunfei Ma et al. [43] introduced a homemade phase transfer reagent (adenosine 5′-monophosphate, AMP) and silane coupling agent (3-mercaptopropyltrimethoxysilane, MPS) into the Stöber system. Oil-soluble (initial CdSe/CdS/ZnS QDs), alcohol-soluble (AMP-QDs), and water soluble (hydrolysis of TEOS around the QDs) were the whole progress of solubility change. QD@SiO_2_ had the same photoluminescence efficiency (50–65%) as the initial one. Wider pH range (pH 4–8 to 2–13), improved stability in electrolyte, better thermal stability, and increased biocompatibility in Hela cells were the advantages of the QD@SiO_2_.

**Fig. 10:**
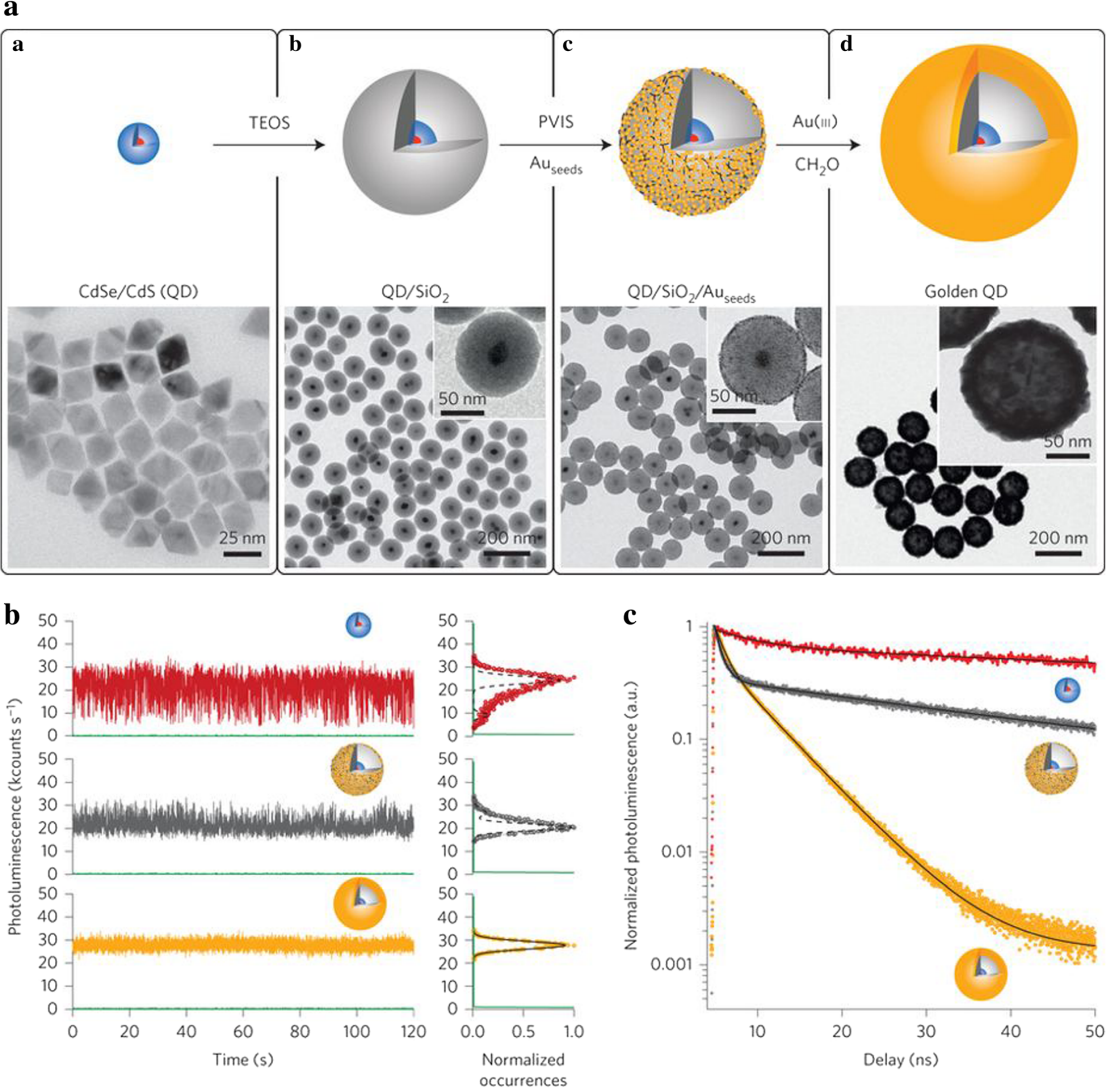
**a** Schematic of quantum dot/SiO_2_/Au hybrid (golden QD) and the TEM images of each stages (CdSe/CdS QDs, QD/SiO_2_ QD/SiO_2_/Au_seed_ and golden QDs). **b** The change of photoluminescence intensity with time. Red for CdSe/CdS, gray for QD/SiO_2_/Au_seed_, and orange for golden QDs. **c** Photoluminescence decay curves of three nanoparticles in (**b**) [45]

To ensure the stability of QDs in optical devices, it is necessary to reduce the effect of blinking. Blinking is a phenomenon with a random intermittent luminescence which affects the stability of the QDs optical devices [62]. To reduce the effect of blinking, Botao Ji et al. [45] produced CdSe/CdS QDs as the core materials and encapsulated these QDs into silica shell based on the displacement of the initial hydrophobic ligands by reverse microemulsion method. And the QDs further modified by an Au layer on the surface of silica with poly (1-vinylimidazole-co-vinyltrimethoxysilane) (PVIS) as the silane coupling agent. The nano-sized gold shell acted as a plasmon resonator that gave the QDs enhanced optical states density. The QDs’ properties can be preserved regardless of drastic changes in the local environments because of the hybrid layer. As a result, the QDs’ photostability increased. The QD fluorescence lifetimes reduced from 123 to 20 ns after gold coated. The golden QDs showed efficient multiexciton emission and its neutral photoluminescence intensity was higher than that of QDs. The results of the stability test were shown in Fig. 10. What is more, the photoluminescence intensity could keep stable for several hours (even 24 h). The luminescence stability test showed that the luminescence of bare QDs would have a dramatic fall after only 1 h. Silica layer improved the performance of QDs slightly but it provided the suitable interval for the next Au layer to show the plasma enhanced effect.

## Synthetic Methods of LSNs

**Fig. 11:**
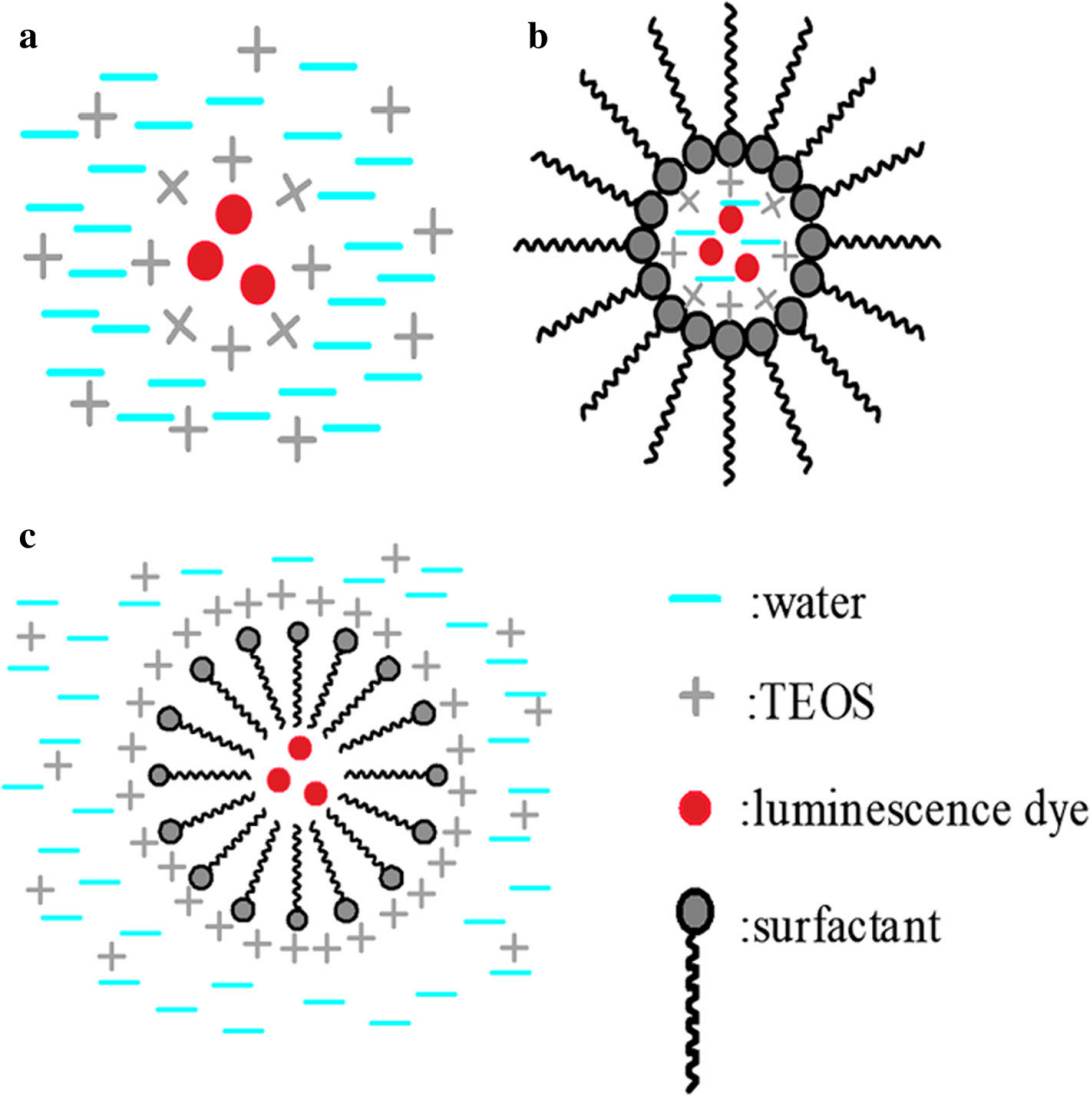
The shematic illustrations of different LSNs with different methods. **a** For Stöber method. **b** For reverse microemulsion method, **c** For direct micelles assistant method

For the fabrication of LSNs, selection of phosphors and design of synthetic routes are the core contents. Phosphors determine the emission range of LSNs and synthetic routes establish their structures and functions. All the synthetic routes of LSNs are based on the silica. Sol-gel method, reverse microemulsion method, and direct micelles assistant method are three major approaches to obtain homogeneous and regular silica spheres which have been used in LSNs. Figure 11 is the schematic diagram of the mentioned methods.

### Sol-Gel Method

Sol-gel method, also called Stöber method, is a convenient and feasible method to obtain monodispersed silica nanospheres. It is ideal to synthesize silica nanospheres since Stöber [63] carefully studied the synthesis of size-specific silica spheres in the range of 50 nm–2 μm with alkoxysilane hydrolyzing under ammonia catalysis. Homogeneous silica spheres with different sizes (10 to several hundreds of nanometers) can be easily obtained by controlling the synthesis conditions such as the ethanol-to-water ratio, the amount of ammonia, and the temperature via sol-gel method. Using Stöber method, Van Blaaderen and A. Vrij Langmuir successfully synthesized dye (FITC)-doped silica by adding (APS) in the reaction system [21]. With the amine group from APS, silica spheres caught FITC easily as showed in Fig. 11a. So far, in addition to dyes, many other materials have been linked to silica via Stöber method. Luis M. Liz-Marzan et al. improved the Stöber method and synthesized gold-silica core-shell particles using (3-aminopropyl)-trimethoxysilane (APTS) as the surfactant [64]. Combined with the gold core, APTES provides chemical bond bridging for silica encapsulation. Alkaline condition leads to homogeneous silica spheres as a popular Stöber system and acid catalyzed hydrolysis of alkoxysilane is also a feasible method to encapsulate luminescent dyes into silica [65].

**Fig. 12:**
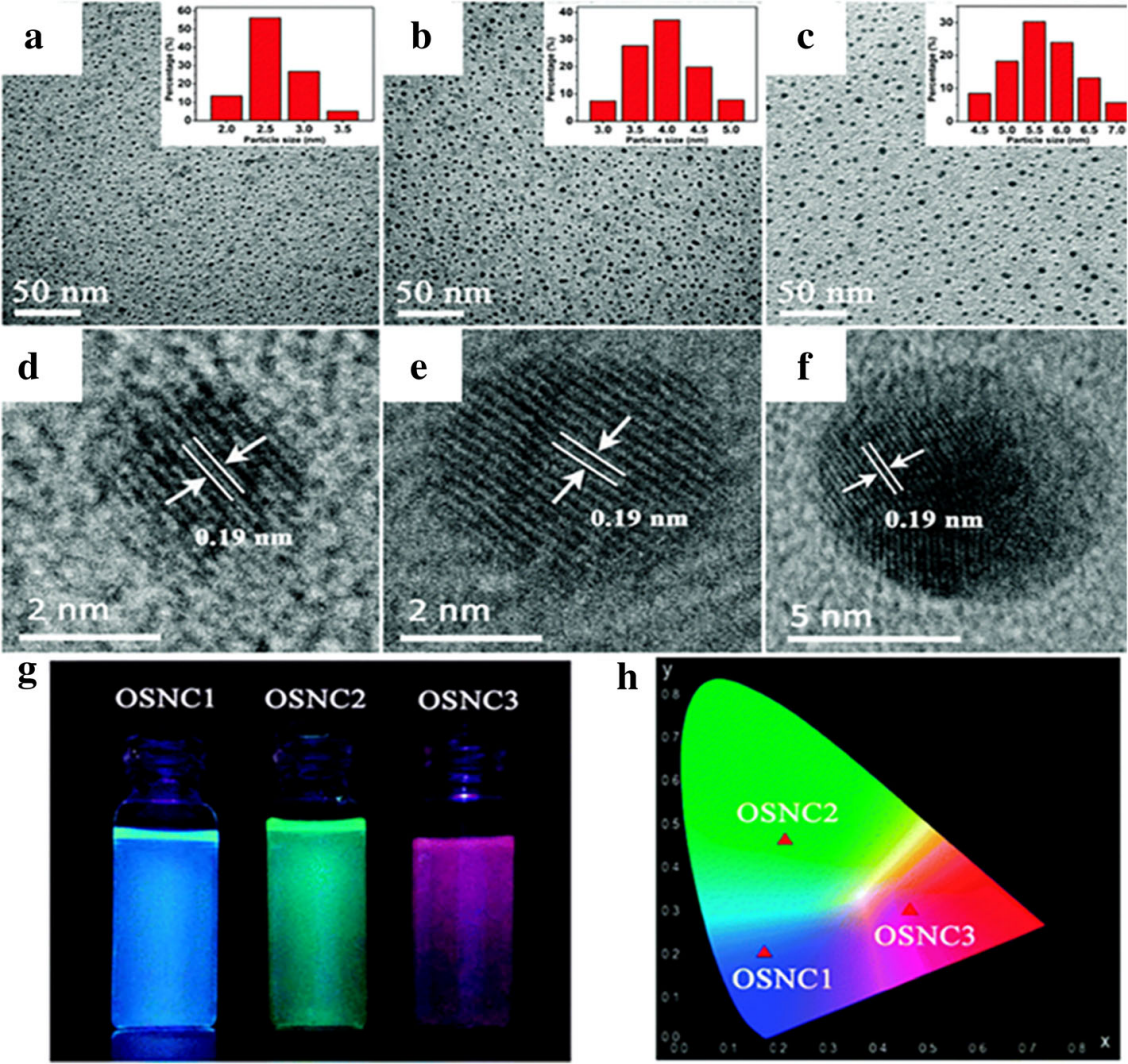
Characterizations of OSNCs: **a**–**c** as TEM images and **d**–**f** as high resolution transmission electron microscopy (HRTEM) images. **g** The photographs of OSNC samples subject to UV-light illumination. **h** The distribution on the Commission Internationale de l’Eclairage (CIE) chromaticity diagram [50]

A new kind of LSNs has been synthesized based on Stöber method. Lingang Yang et al. [50] successfully synthesized crystal silica by Stöber method based on the π-π stacking of vinyl groups. A Stöber progress with vinyltriethoxysilane (VTES) as the precursor, neutralization with hydrochloric acid, vacuum distillation to remove the solvent, and extraction using tetrahydrofuran are the whole procedures of organosilica nanocrystals (OSNCs). Three OSNCs had been synthesized with the same crystal structure but in different sizes as shown in Fig. 12d–f. The sizes of organosilica nanocrystals (OSNCs) are gradually increasing because of the increase of VTES. As a result, they showed different luminescent properties as showed for Fig. 12g, h (blue, green, and red under UV light). OSNCs were characterized to possess good photo-stability and pH stability. The epitaxial growth of vinyl groups in diamond cubic crystal structure are presented due to the π-π stacking. The orderly stacked vinyl groups finally form a large π conjugated system with fluorescence following the quantum confinement. These OSNCs had great potential on the optical fields owing to the characteristics of silica which provided a new approach to get self-luminescence silica materials.

### Reverse Microemulsion Method

**Fig. 13:**
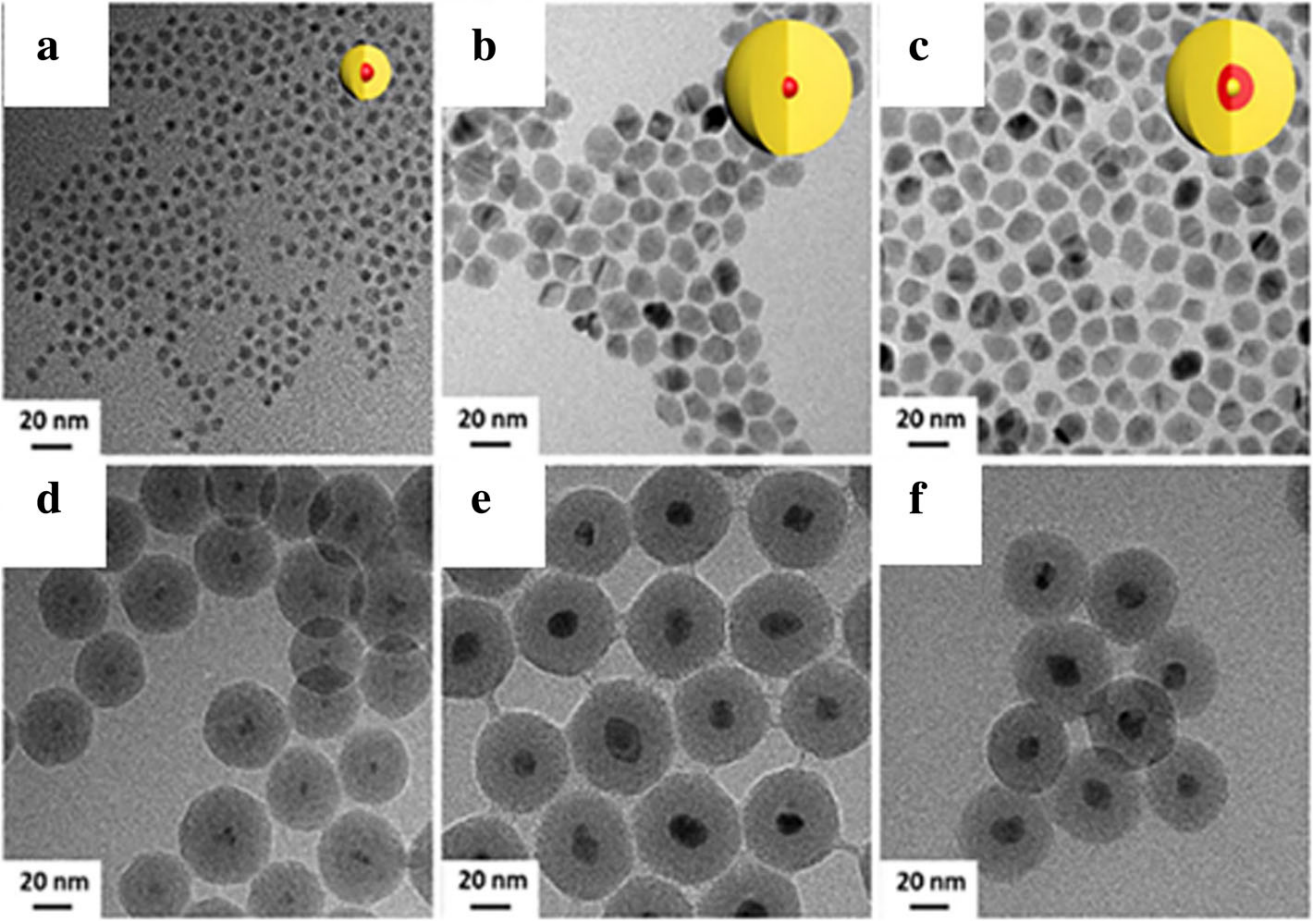
TEM images of CdSe/CdS core/shell QDs with CdS thin shell (**a**) and after coating with silica CdSe/CdS@SiO_2_ (**d**); CdSe/CdS core/shell QDs with CdS shell (**b**) and after coating with silica CdSe/CdS@SiO_2_ (**e**); CdS/CdSe/CdS core/shell QDs (**c**) and after coating with silica CdS/CdSe/CdS@SiO_2_ (**f**) [46]

Stöber method is a simple and convenient method to synthesize LSNs, but reaction conditions and initial particles that are not controlled place restrictions on the luminescent dyes. To overcome such limitations, Bagwe and Khilar [66] introduced water-in-oil microemulsion system [67] during the synthesis of silver coated with silica nanocomposites (Fig. 11b). The initial alkaline aqueous solution of silver nanoparticles with TEOS was encapsulated in the water drop using surfactants. The hydrolysis progress of TEOS was the same as that of Stöber method. But the whole progress was restricted into water droplets enclosed by surfactants which led to a well-controlled system and monodispersed silica nanoparticles. The size of silica was well controlled by selecting different surfactants, solvents, and changing the ratio of surfactant to water. When the fluorophores are hydrosoluble, it is easy to form a homogeneous silica layer on the surface within the molecules in the droplet. Nianfang Wang et al. [46] synthesized luminescent silica-coated CdS/CdSe/CdS nanoparticles via reverse microemulsion method. Figure 13 showed the TEM images of the synthetic QDs and QDs@SiO_2_. The protected QDs showed excellent acid and thermal stability. It provided possibility for further modification to meet special requirements for applications.

### Direct Micelles Assistant Method

Reverse microemulsion method require the water-soluble luminescence dyes. Inversely, liposoluble initial micelles are the major features of direct micelle method, and the hydrolysis progress takes place around of the micelles (Fig. 11c). A precursor is indispensable for the agglomeration of silica. As a common progress, the luminescent dye is modified with the silane coupling agent, such as APS, to form the assistant micelles. The initial modified micelles ensure that the TEOS condensation occurs around them. Using Rhodamine B conjugated to APTES as the original micelle, Kumar et al. [26] successfully synthesized Rhodamine-conjugated organically modified silica nanoparticles in oil in water system and modified them with different function groups (such as sulfhydryl, amino, and carbonyl) which can be used as cell fluorescence probe.

The role of the surfactant is not only reflected in the silica synthesis but also in the synthesis of mesoporous silica. A common method of synthesizing mesoporous silica is calcination. Large specific surface area and modifiable surfaces make the mesoporous silica nanoparticles perfect carriers. In addition to the known application value in the field of medical drug loading, it also has important application prospects in the field of loading phosphors. Li Wang et al. [68] mixed up CDs with hollow mesoporous silica microspheres with good photochemical stability which can be used for oxygen detection in the whole range. Mesoporous structure makes them unique. Bin Xie et al. [69] incorporated the CdSe/ZnS core-shell QDs into mesoporous silica microspheres by a swelling and evaporation method. Coated with a mesoporous silica layer on the surface of Gd_2_O_3_:Eu phosphors via modified Stöber method is also feasible according to the Ali Aldalbahi et al. [70]. Because of the encapsulation of silica, the modified Gd_2_O_3_:Eu nanoparticles showed excellent solubility and biocompatibility.

### Other Methods

There are also other methods to synthesize LSNs such as chemical vapor deposition (CVD) [71], hydrothermal method [51], and amino acid-catalyzed seed regrowth technique [72, 73].

Lianzhen Cao et al. [71] synthesized SiC/SiO_2_ by CVD and thermal annealing processes. Si was used to coat on the SiC core by thermal CVD and then SiO_2_ shell was obtained after oxidizing. The annealed SiC/SiO_2_ nanoparticles showed narrow luminescence in the blue-green region. The synthetic method provided a new way to synthesize core-shell nanomaterials.

Chandra et al. [51] synthesized smaller fluorescent silica nanoparticles (1 to 2 nm) with silicon tetrabromide (SiBr_4_) and APTS. Heating to 200 °C in an autoclave was the core step of the whole reaction. The final products were obtained after further purification including dialysis and centrifugation. The silica nanoparticles emitted bright blue luminescence with a photoluminescence quantum yield around 34%. It was non-photobleaching and biocompatible at the same time.

Surface modification makes the LSNs more tunable for complex application [74]. Silane coupling agents are the most common chemical methods as it mentions before. Abundant hydroxyl groups provide reaction sites for further modifications. Junqiang Wang et al. synthesized silica modified CeO_2_ ammonia sensor with high gas response due to hydroxyl groups [75]. After hydrolysis and condensation, silane coupling agents with different function groups bond on the surface of silica. Superhydrophobic silica was synthesized with the condensation of VTES (-CH=CH_2_) [76]. Ming Ma et al. grafted PEGMA and DMEAA on the surface by RAFT polymerization based on the -NH_2_ of APTS [77]. Surface modification can enhance their adaptability in complex environments and get improved luminescence properties with appropriate materials.

Among these methods, there are two main ideas to fabricate LSNs, namely the luminescent dyes are added directly into the reaction system when the silica resources start hydrolyzing, and that the luminescent dyes are established chemical bond with silica by other reagents such as silane coupling agents, either before or after silica network set up. It is necessary to select and design an appropriate synthetic route for LSNs with specific structures.

## Applications of Luminescent Silica Nanoparticles

Light is the most intuitive tool for people to recognize the world. Luminescent materials with special emission can be directly used in many ways such as lighting, display, and so on. At the same time, changes in fluorescence intensity can reflect some important information. Compared with separate luminescent dyes, LSNs have improved performances in applications, since silica provides a stable matrix for the luminescent dye. It provides an effective way for multifunction at the same time [6]. LSNs with multifunction and tunable surface have great application prospects and development potential in biology, lighting, and sensors.

### Biolabeling and Medicine

LSNs have great application value in biology. Non-toxicity is a fundamental requirement for medical field, especially in vivo [78]. The fact that the common luminescent dyes are often toxic limits their clinical application [79]. Silica, a favorite non-toxic modified material, is a good solution to elimination of toxicity. Toxicity of silica nanoparticles (20–200 nm) were also carefully studied by In-Yong Kim et al. [80]. Size, dose, and cell type-dependent cytotoxicity were the issues in their research. Although high dose can cause a disproportionate decrease in cell viability, the silica nanospheres with 60 nm showed their good biocompatibility up to 10 μg/ml. Different cells had different tolerance to silica nanoparticles which indicated that it was necessary to have substantial tests before clinical tests. Although inhalation of silica particles can cause acute and chronic diseases including silicosis [81], silica still has potential in biological application at the nanoscale. The toxicity of luminescent silica nanoparticles to living cells was studied in detail by Yuhui Jin et al. [38]. From the DNA level to the cell level, the toxicity of RuBpy-doped LSNs were carefully tested. At a certain concentration, the results showed that the dye-doped luminescent silica nanoparticles were non-toxic to the targeted DNA and cells, which indicate that LSNs are a good solution to the non-toxic modification. Xiqi Zhang et al. [27] encapsulated AIE dye (An18, derivatized from 9, 10-distyrylanthracene with an alkoxyl endgroup) into the silica nanoparticles via a one-pot modified Stöber method. Coated with silica lead to an enhanced fluorescence intensity, good water solubility, and non-toxicity to living cells which made the An18-SiO_2_ NPs had a potential for biomedical application.

**Fig. 14:**
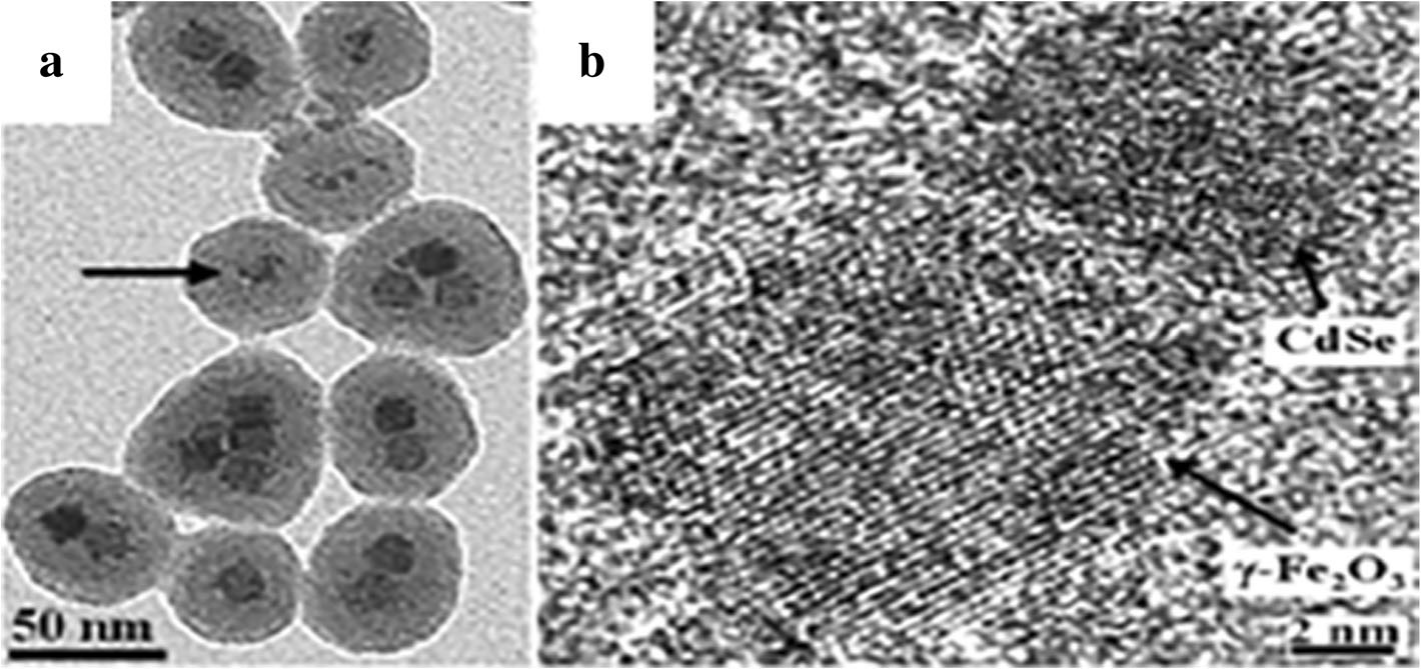
**a** TEM image and **b** HRTEM image of SiO_2_/MP-QD nanoparticles [48]

**Fig. 15:**
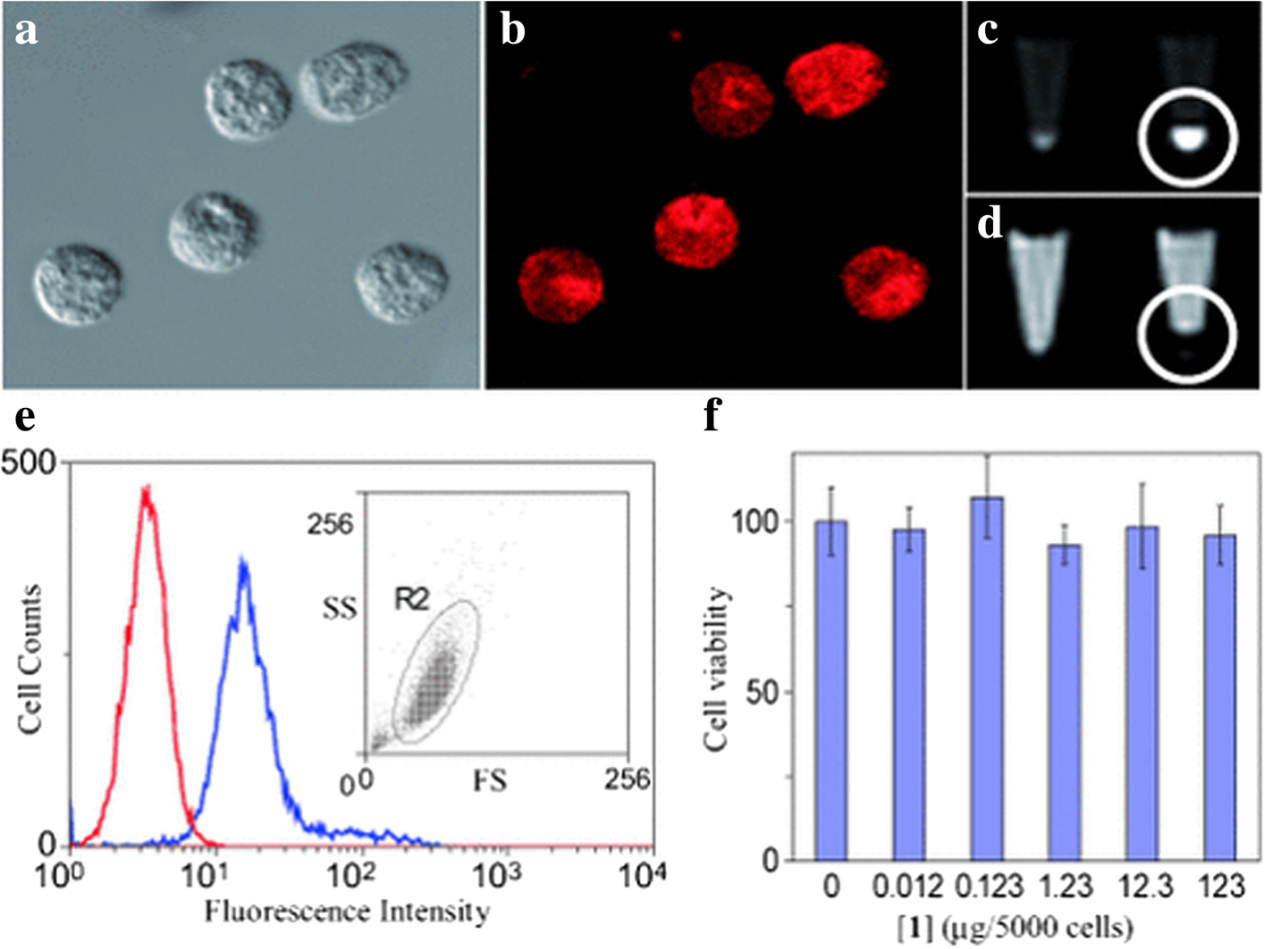
The imaging results of monocyte cells with **a** optical microscopic, **b** laser scanning confocal fluorescence, **c**, **d** the images of monocyte cells with MR: left for unlabeled monocyte cells and right for hybrid silica nanoparticles labeled monocyte cells, **e** flow cytometric results of blank and hybrid silica nanoparticles-labeled monocyte cells, and **f** the cell viability with different amount of hybrid silica nanoparticles [39]

LSNs have great application value in diagnosis and biolabeling. For hybrid imaging contrast agents, Dong Kee Yi et al. [48] combined magnetic particles (MPs) Fe_2_O_3_ with QDs (CdSe) and encapsulated them in silica shell by reverse microemulsion method. The nanostructures of MPs with QDs are clearly showed in Fig. 14. Magnetic resonance imaging (MRI) is an effective method for disease detection, especially for cancer. The advantages of feasible usage, low cost, and accurate diagnosis make it more popular as a diagnostic tool [7]. The nanocomposites can be used as both optical and MRI contrast agents. It is worth mentioning that the presence of CdSe increased the effective magnetic anisotropy of the γ-Fe_2_O_3_-containg particles. This is a good attempt, but the low quantum yield (SiO_2_/MP-QD 1.1% to CdSe 11.4%) limits the actual effect. Willam J. Rieter et al. [39] also synthesized the same multifunctional nanocomposites. What is different is that [Ru (bpy)_3_] Cl_2_ was chosen as the luminescent core and the paramagnetic Gd complex was coated on the luminescent core by water-in-oil reverse microemulsion method. The nanocomposites were finally embedded in silica in the same way. The results of Fig. 15 proved that hybrid silica nanoparticles had good optical and MRI performances in biological imaging. Mesoporous silica nanospheres doped with europium (Eu-MSN) were obtained by Mengchao Shi et al. [32]. Nanoscale size (280–300 nm) and fluorescent property were the basic for an ideal biolabeling material. They found that Eu-MSNs had a positive influence on osteogenesis and angiogenesis-induction. By promoting proper response of macrophages and the expression of relevant genes, the defect of bone replaced by new bone and the healing process of skin wound can accelerate with Eu-MSNs. Besides the function of biolabeling, the LSNs showed the potential in tissue repair. LSNs can achieve the target binding effect by modifying the special group. In Duarte’s work [33], organosilane Bpy-Si was chosen as a ligand of Eu complex for the further reaction with silica. SiO_2_-[Eu (TTA)_3_(Bpy-Si)] nanoparticles were obtained with a uniform size (28 ± 2 nm). With a further modification of an amino acid spacer and an anchor group (anti-*Escherichia coli*, IgG1), the functionalized silica had the specific bonding with *E*. *coli* bacteria. It was easy to get the distribution of *E*. *coli* bacteria with luminescence. The bio-multifunction of LSNs was also carefully studied by Laranjeira et al. [82]. Gadolinium (Gd) composites with unique magnetic properties have potential in MRI contrast agents but Gd3+ ions are toxic in humans especially in kidneys and pancreas. GdOHCO_3_ nanoparticles were chosen as the MRI contrast core and coated with silica layer via Stöber method. With the silica coating, the Gd composite (SiGdOHCO_3_) had the same brightness of MRI contrast images but no degradation at designed pH values (5.5, 6.0, and 7.4). And SiGdOHCO_3_ had little effect on human fibroblasts according to the cell proliferation assay which indicated an excellent biocompatibility. Silica provides a more stable environment and further possible modification for GdOHCO_3_ without affecting MRI performance. By diverse micelles method, Atabaev et al. [83] synthesized Gd_2_O_3_:Tb^3+^,Eu^3+^@SiO_2_ nanoparticles which can be used as both MRI contrast and fluorescence agents in vivo. The above two examples perfectly reflected the role of LSNs in multifunction with the silica platform.

LSNs have great application value in drug delivery. Hongmin Chen synthesized luminescent mesoporous silica nanoparticles biofunctionalized by targeting motifs, which makes them applicable in drug delivery [47]. They first prepared APS-containing mesoporous silica particles, and subjected the products to calcination at 400 for 2 h. They synthesized mesoporous silica by the help of cetyltrimethyl ammonium bromide (CTAB). There were luminescent carbon dots in the silica matrix after calcination. The fluorescence intensity was at the maximum when the particles were excited at 380 nm. The target selectivity of FL-SiO_2_ was achieved by surface modification of RGD peptide with the help of APS. They also studied the RGD-FL-SiO_2_ loading and release of doxorubicin (Dox). After calcination, fluorescent mesoporous silica (FL-SiO_2_) can still load Dox effectively. The porous structure was not affected by calcination. They found that RGD-FL-SiO_2_ had good luminescent effect especially around the blood vessels of tumor in vivo imaging studies. Integrin α_v_β_3_ of the tumor was the key of the interactions. Although there are many excellent attempts to apply LSNs to medicine but less successful clinical tests in human beings means that there is still a long way to go for the real medicine applications.

### Light-Emitting Devices

Due to their special emitting features, LSNs also play a vital role in light emission fields including the field emission- and liquid crystal-based display technologies [84]. WLEDs have received recent attention for their broad applications including general illumination and displays. Tunable color, high color purity, and luminescence efficiency are in line with the requirements of light-emitting diodes (LEDs) [85]. Quantum dot-based light-emitting diodes (QD-LEDs) have demonstrated recently, and may offer many advantages over conventional LED and organic light-emitting diodes (OLEDs) technologies in terms of color purity, stability, and production cost, while still achieving similar levels of efficiency. In order to improve the performances of polymer dots (Pdots) as WLED phosphors, Kaiwen Chang et al. [49] introduced some Pdots with different emission wavelength into the Stöber system to get encapsulated. The silica-encapsulated Pdots showed the same luminescence properties but markedly enhanced photostability.

**Fig. 16:**
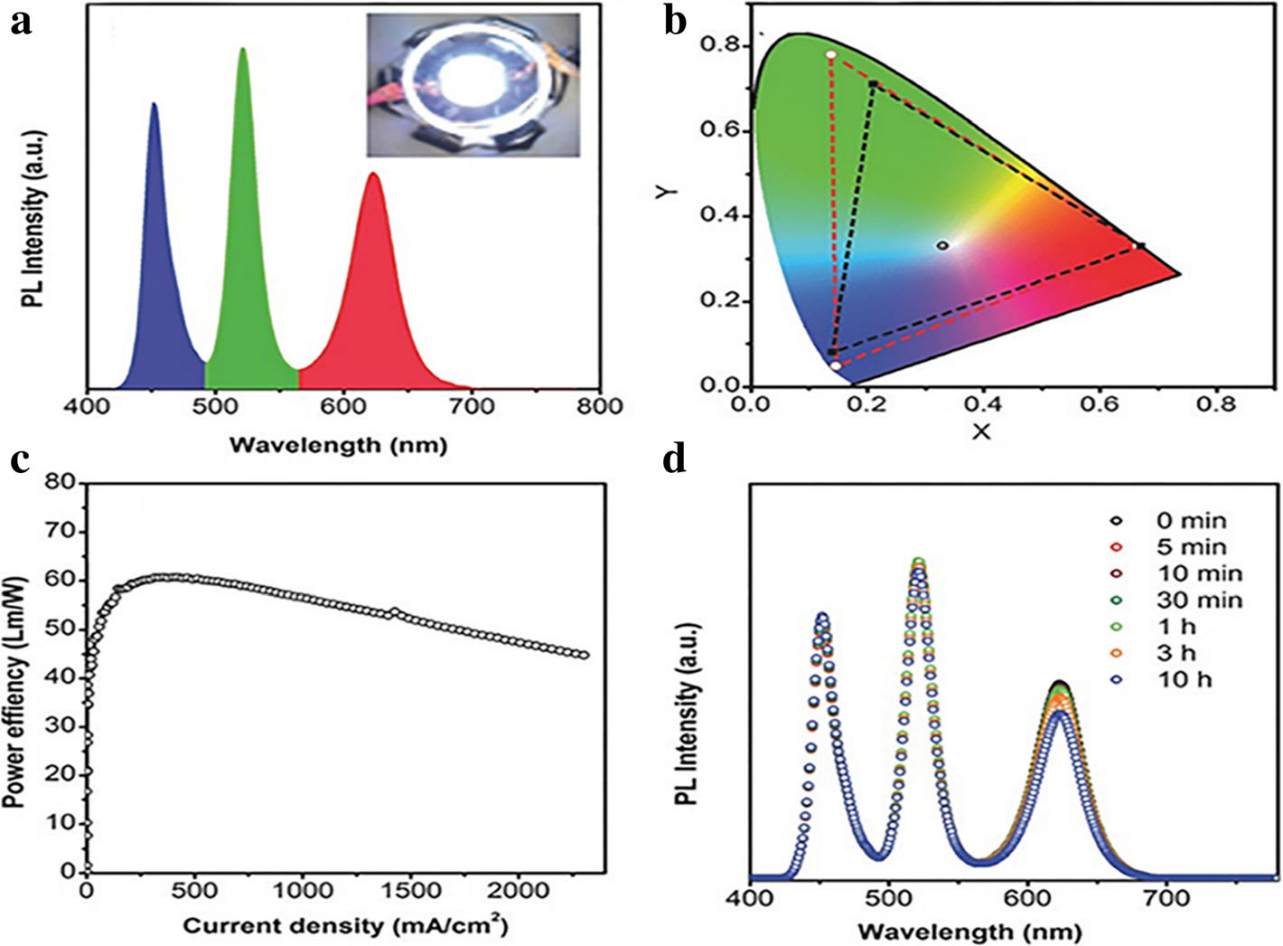
The optical performances of the WLED: **a** the emission spectra, **b** the CIE color coordinates and the color triangle of WLED (red dashed line) with the NTSC TV standard (black dashed line), **c** the power efficiency, and **d** emission spectra after working for a while [34]

To reduce the manufacturing complexity required for achieving full-color displays, it is more desirable to use a common device structure to achieve high efficiency for three primary colors (blue, green, and red). QDs have been widely used in display field because of its unique luminescent properties, such as high luminescent intensity, narrow emission spectra, and tunable emission. Chun Sun et al. [34] synthesized the perovskite QDs, CsPbBr_3_, as the light-emitting core of WLEDS. Only the perovskite QDs are not enough for a LED device since photostability and stability are necessary for an optical device under long-time work and elevated temperature. There are anion-exchange reactions between different halide QD nanoparticles which would widen the narrow emission spectrum. QD/silica composite were fabricated in APS to avoid oxidation and decomposition. So they used APTES as the QDs’ capping agent and improved the silica coating process to avoid the decomposition of the QDs. Green and red QD/silica composites were synthesized and a WLED was obtained by the combination of the composites with a blue LED chip. The WLED had good performances with great air stability as depicted in Fig. 16.

**Fig. 17:**
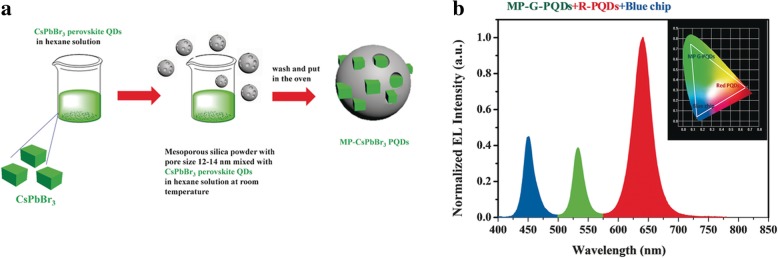
**a** The formation progress of MP-CsPbBr3PQDs. **b** The luminescence intensity and the color triangle of WLED [35]

**Fig. 18:**
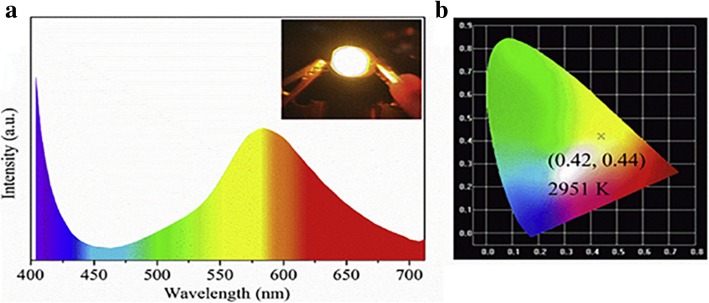
The performance of WLED showed as **a** the emission spectrum and **b** for CIE chromaticity and CCT [36]

LSNs can keep good dispersion, brightness, and photostability of QDs. Hung-Chia Wang et al. [35] provided a new composite method for QDs and silica (Fig. 17). By mixing the QDs with mesoporous silica powder of which pore size was bigger than that of QDs in non-polar solution, mesoporous silica green PQD nanocomposite was obtained after washing and drying. The quantum dot showed better thermal stability and photostability after composited with silica. On the other hand, QDs are a typical kind of aggregation-caused quenching (ACQ) nanoparticles, which means that it is necessary to keep a good dispersion to get a good brightness and photostability. Kai Jiang et al. [86] synthesized carbon dots with red, green, and blue luminescence with phenylenediamines as precursors to enhance luminescence properties as solution and poly (vinylalcohol) (PVA) film. But it would exist quenching effect as solid-state CDs which was fatal for LED devices owing to aggregation and the result Förster resonance energy transfer (FRET). To avoid the dispersion and the resulting FRET phenomenon, Junli Wang et al. [36] embedded carbon dots into silica matrix (Fig. 18) by dispersing carbon dots into the *N*-(3-(trimethoxysilyl)propyl) ethylenediamine (KH-792) and heating to form a homogenous CD/silica film. A white LED was fabricated by drying the CD/silica solution on the inner wall. By the assistant of silica, CDs were well dispersed with an appropriate distance without quenching which improve the performance as powders. Figure 18 showed the emission spectra and performance in WLED. And the CIE coordinates (0.44, 0.42) and correlated color temperature (CCT) (2951 K) suggested that it was suitable for indoor illumination.

### Sensors

Luminescent silica showed the excellent performances on static luminescent materials, such as biolabeling and WLED phosphors. All these were based on their unique and stable optical properties. When it came to dynamic luminescent materials, LSNs also display the same wonder [9]. The luminescent sensors of pH [28], ions [87], and temperature [40] are following as representatives.

pH value have great influence on the luminescence intensity which inspires luminescent pH sensor. In the same principle as ref. [22], Atabaev et al. synthesized the same ratiometric pH sensor [28]. FITC was chosen as the pH-dependent luminescence dye and Y_2_O_3_:Eu^3+^ as pH stable dye. With the Stöber coating of silica, Y_2_O_3_:Eu^3+^@SiO_2_ with FITC composite NPs were successfully synthesized. The change of pH was reflected by the ratio of fluorescence intensity (*I*_FITC_/*I*_Y2O3:Eu3+_). The standard dye led to a less influence of concentration and a more accurate result.

**Fig. 19:**
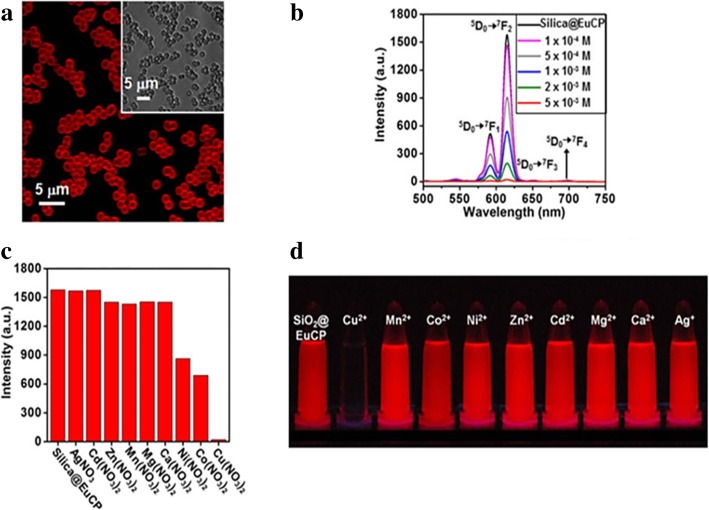
**a** Confocal microscopy and OM (inset) images of silica@EuCP microspheres. **b** Luminescence spectra with different Cu (NO_3_)_2_ in MeCN; luminescence intensity changes (**c**) and photograph (**d**) with different metal ion solutions (5 mM) [37]

**Fig. 20:**
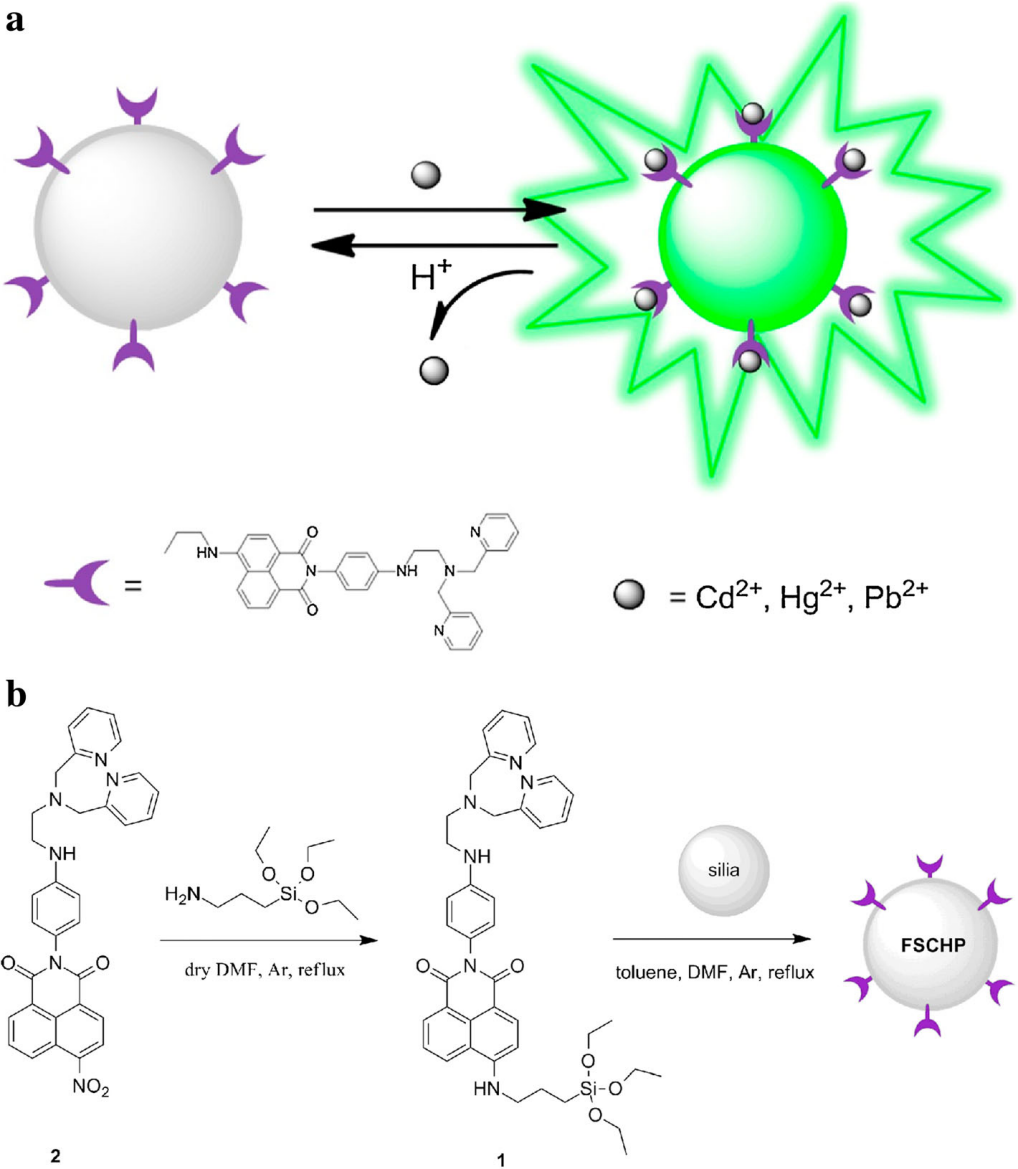
The formation and sensing progress scheme of sensitive fluorescent sensor (FSCHP) [29]

LSNs can also be used as ions sensors. Based on the changes of luminescence intensity with the measured physical quantity, LSNs have been applied to many sensor fields by the environment-dependent effect of the luminescence. Quenching effect is an effective detective tool to detect the changes of quenching factors such as ions and pH value with external quenching mechanisms such as FRET and photoinduced electron-transfer (PET) [9]. Sensors for metal ions are important fields whether in cells or open system. Won Cho et al. [37] synthesized europium (III) coordination polymer (EuCP) and found the specific quenching effect of Cu^2+^ (Fig. 19). In view of this fact, they synthesized silica@EuCP microsphere which have the same sensitivity on Cu^2+^ with less mass of europium. As an auxiliary material, silica can effectively reduce the amount of sensor materials. Both of them have their unique situations. Besides quenching effect, there are some different effects which can be used in the fields of sensors. 2,2-Dipicolylamine (DPA) and its derivatives have good affinity to heavy ions. And enhanced luminescence effect would happen after chelated with heavy ions. Yu Ding et al. [29] modified silica spheres with *N*,*N*′-bis (pyridine-2-ylmethyl)ethane-1,2-diamine (Fig. 20). The concentration of heavy ions (Cd^2+^ Hg^2+^ and Pb^2+^) in samples can be determined by the change of fluorescence intensity. The test in real water samples and simulated biological samples confirmed the heavy metal ions-binding ability and the detection which has application prospects in the water monitoring and so on.

**Fig. 21:**
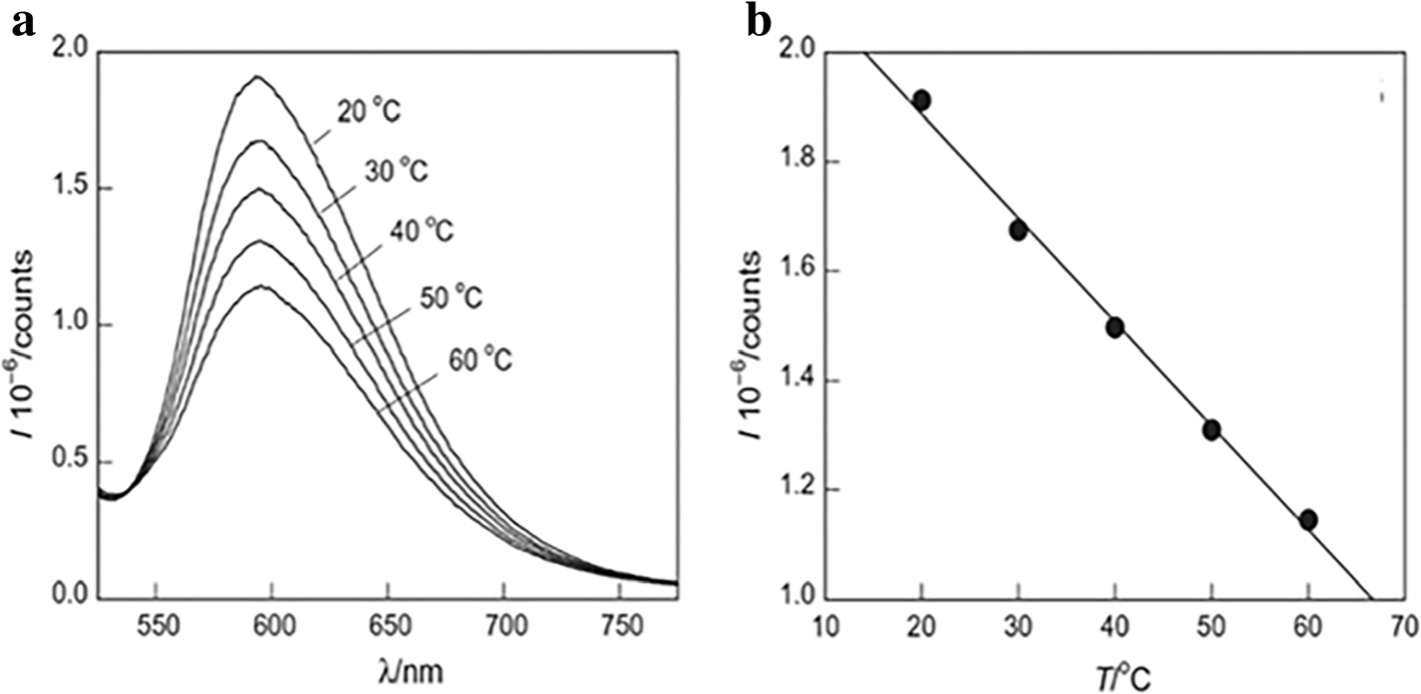
**a** PL spectra of Ru (bpy)_3_@SiO_2_ under different temperature. **b** The peak intensity changes as a function of temperature [40]

**Fig. 22:**
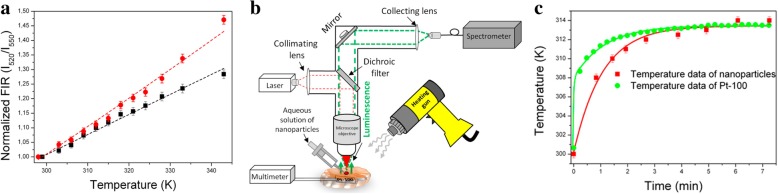
**a** *I*_520_/*I*_550_ with different temperature for Er, Yb:GdVO_4_ and Er, Yb:GdVO_4_@SiO_2_. **b** The sketch map for the ex vivo temperature determination experiment. **c** The results of the temporal evolution of temperature for the Er, Yb:GdVO_4_@SiO_2_ and a thermoresistor Pt-100 [41]

Temperature sensors are also important applications of LSNs. Temperature is a basic variable in most science fields. The temperature dependence of radiative and non-radiative transition rates is the core content of temperature sensing which makes it possible for luminescence temperature sensing, with the contactless and large-scale advantages [9]. However, in order to be applied in practice, their stability is crucial as the environment of application is more complex than of that of experiment condition. Silica is an ideal matrix to improve their performance for application. Mirenda et al. [40] synthesized silica as the core and then TEOS was hydrolyzed with Ru (bpy)_3_Cl_2_ to form the Ru (bpy)_3_@SiO_2_ NPs. The emission spectra of Ru (bpy)_3_@SiO_2_ NPs (Fig. 21) showed that the intensity of Ru (bpy)_3_@SiO_2_ NPs decreased linearly as the temperature rising as the result of the activated non-radiative ^3^d-d state (20–60 °C, λ_exc_ = 463 nm). The polyethyleneimine (PEI)-modified glass with Ru (bpy)_3_@SiO_2_ NPs showed the same trend as the NPs which proved that the potential as the temperature sensing. With cycling the temperature between 20 and 60 °C, the relative slope decreased until the seventh cycle which meant that it is necessary to condition to obtain the stable sensing materials. The influence of temperature on probes is complicated. So it is necessary to research the temperature-dependent luminescence of the probes to know how to apply it into temperature sensors. Temperature is a fundamental variable that governs diverse intracellular chemical and physical interactions in the life cycle of biological cells. The change of temperature reflects the level of cell metabolism. GdVO_4_ co-doped with Er^3+^ (1 mol%) and Yb^3+^ (1 mol%) has the potential to apply as the temperature sensor. To improve their performance as temperature sensor, Savchuk et al. [41] coated silica shell on the nanoparticles surface by Stöber method. The fluorescence intensity ratio (FIR) of Er, Yb:GdVO_4_, *I*_520_/*I*_550_, had a certain linear relationship with temperature in the range from 297 to 343 K after excitation at 980 nm. And the probes got enhanced thermal sensitivity, high thermal resolution and good stability in different solvents. And the result of the ex vivo experiment to monitor temperature evolution with the special sensor showed in Fig. 22 proved that Er, Yb:GdVO_4_@SiO_2_ core-shell nanoparticles had a good thermal resolution as the temperature sensor in biomedical applications.

## Conclusion

In this article, LSNs with various functions demonstrate that silica is an ideal host material for luminescent dyes. The visualization of related parameters is the most special feature of luminescent dyes. Various luminescent materials have their own advantages but there are still some defects which limit their applications. Improved brightness, photostability, and thermal stability are the advantages of LSNs with the protection of silica. At the same time, it provides phosphors with a versatile platform which makes it possible to become multifunctional and specially modified. Excellent performance, adjustable adaptability, and potential versatility broaden the applications of fluorescent materials. LSNs have great potential in many unmentioned fields such as solar cells and photocatalysts. However, there is still a long way to apply LSNs to the actual species. Poor selectivity and low signal-to-noise ratio in complex conditions are factors that constrain LSNs for the practical applications which need to be further studied. Defined distances between phosphors and LSPR metal deserve more investigations to get the positive effect. Many new luminescent materials with excellent luminescence properties have been developed which means that it is necessary to improve the traditional synthetic methods to obtain LSNs. Silica is a traditional modified material but LSNs still have great potential for development.

## Abbreviations

ACQ: Aggregation-caused quenching
AIEgen: Aggregation-induced emission luminogens
AMP: Adenosine 5′-monophosphate
An18: An aggregation-induced emission-based organic fluorogen derivatized from 9,10-distyrylanthracene with alkoxyl endgroup
APS: (3-Aminopropyl)triethoxysilane
APTES: 3-Aminopropyltriethoxysilane
APTS: (3-Aminopropyl)trimethoxysilane
B: Blue
BAM: Bio-anchored membrane
CCT: Corresponding correlated color temperature
CDs: Carbon dots
CDSP: Carbon dot-silica- phosphor composite
CIE: Commission Internationale de l’Eclairage
CLSM: Confocal laser scanning microscope
CRI: Color rendering index
CTAB: Cetyltrimethyl ammonium bromide
CVD: Chemical vapor deposition
DDT: 1-Dodecanethiol
Dox: Doxorubicin
DPA: 2,2-Dipicolylamine
F127: Poly (ethylene glycol)-block-poly (propylene glycol)-block-poly (ethylene glycol)
FIR: Fluorescence intensity ratio
FITC: Fluorescein isothiocyanate
FL-SiO_2_: Fluorescent mesoporous silica
FRET: Förster resonance energy transfer
FSCHP: Sensitive fluorescent sensor
FSNP: Fluorescent silica nanoparticle
G: Green
H: The ratio of water/TEOS
HPTS: 8-Hydroxypyrene-1,3,6-tresulfonic acid
HRTEM: High resolution transmission electron microscopy
IgG1: Anti-Escherichia coli
KH-792: N-(3-(trimethoxysiyl)propyl)ethylenediamine
LEDs: Ligh-emitting diodes
LSN: Luminescent silica nanoparticle
LSPR: Local surface plasmon resonance
MPS: 3-Mercaptopropyltrimethoxysilane
MPs: Magnetic particles
MQDs: Magnetic quantum dots
MRI: Magnetic resonance imaging
MTT: Methyl tetrazolium
NIR: Near-infrared
NTSC: National Television System Committee
OLEDs: Organic light-emitting diodes
OSNC: Organosilica nanocrystal
OTES: n-Octyltriethoxysilane
PBS: Phosphate-buffered saline
Pdots: Polymer dots
PEI: Polyethyleneimine
PET: Photoinduced electron transfer
PVA: Poly (vinylalcohol)
PVIS: Poly (1-vinylimidazole-co-vinyltrimethoxysilane)
QD655: A kind of commercial quantum dots
QD-LEDs: Quantum dot-based light-emitting diodes
QDs: Quantum dots
R: Red
R: The ratio of water/surfactant
RBL-2H3: Rat basophilic leukemia mast cells
SEM: Scanning electron microscope
TEM: Transmission electron microscope
TEOS: Tetraethoxysilane
TPETPAFN: A typical fluorogen consisting of two tetraphenylethylene pendants and an intramolecular charge transfer core
TRITC: Tetramethylrhodamine isothiocyanate
UC: Upconversion
UCNP: Upconversion nanoparticles
UCNPs@SiO2@EuTP: NaGdF4:Yb,Er@SiO2@Eu (TTA)3Phen
UV: Ultraviolet
VTES: Vinyltriethoxysilane
WLED: White light-emitting diode

## References

[1] Ronda CR (2008) Luminescence: from theory to applications. WILEY-VCH, Weinheim

[2] Ansari AA, Labis JP, Aslam Manthrammel M (2017). Designing of luminescent GdPO4:Eu@LaPO4@SiO2 core/shell nanorods: synthesis, structural and luminescence properties. Solid State Sci.

[3] Cantelli A, Battistelli G, Guidetti G, Manzi J, Di Giosia M, Montalti M (2016). Luminescent gold nanoclusters as biocompatible probes for optical imaging and theranostics. Dyes Pigments.

[4] Hu F-l, Shi Y-X, Chen H-H, Lang J-P (2015). A Zn (ii) coordination polymer and its photocycloaddition product: syntheses, structures, selective luminescence sensing of iron (iii) ions and selective absorption of dyes. Dalton Trans.

[5] Liu M, Huang H, Wang K, Xu D, Wan Q, Tian J (2016). Fabrication and biological imaging application of AIE-active luminescent starch based nanoprobes. Carbohydr Polym.

[6] Montalti M, Prodi L, Rampazzo E, Zaccheroni N (2014). Dye-doped silica nanoparticles as luminescent organized systems for nanomedicine. Chem Soc Rev.

[7] Yao J, Yang M, Duan Y (2014). Chemistry, biology, and medicine of fluorescent nanomaterials and related systems: new insights into biosensing, bioimaging, genomics, diagnostics, and therapy. Chem Rev.

[8] Qiao J, Zhao J, Liu Q, Xia Z (2019) Recent advances in solid-state LED phosphors with thermally stable luminescence[J]. J Rare Earths 37(6):565–572

[9] Schäferling Michael (2012). The Art of Fluorescence Imaging with Chemical Sensors. Angewandte Chemie International Edition.

[10] Kitai A (2008) Luminescent materials and application. John Wiley & Sons: Hoboken, NJ

[11] Wang J, Shah ZH, Zhang S, Lu R (2014). Silica-based nanocomposites via reverse microemulsions: classifications, preparations, and applications. Nanoscale..

[12] Lin L-S, Song J, Yang H-H, Chen X (2018). Yolk–Shell nanostructures: design, synthesis, and biomedical applications. Adv Mater.

[13] Piao Y, Burns A, Kim J, Wiesner U, Hyeon T (2008). Designed fabrication of silica-based nanostructured particle systems for nanomedicine applications. Adv Funct Mater.

[14] Ghosh Chaudhuri R, Paria S (2012). Core/shell nanoparticles: classes, properties, synthesis mechanisms, characterization, and applications. Chem Rev.

[15] Hua Z, Shishan W, Jian S (2008). Polymer/silica nanocomposites: preparation, characterization, properties, and applications. Chem Rev.

[16] Burns A, Ow H, Wiesner U (2006). Fluorescent core-shell silica nanoparticles: towards "lab on a particle" architectures for nanobiotechnology. Chem Soc Rev.

[17] Eliseevaa SV, Bünzli JCG (2010). ChemInform abstract: lanthanide luminescence for functional materials and bio-sciences. Cheminform..

[18] Nalwa HS, Miyata S (1997) Nonlinear optics of organic molecules and polymers. CRC Press: Boca Raton, FL

[19] Bajorowicz B, Kobylański MP, Gołąbiewska A, Nadolna J, Zaleska-Medynska A, Malankowska A (2018). Quantum dot-decorated semiconductor micro- and nanoparticles: a review of their synthesis, characterization and application in photocatalysis. Adv Colloid Interf Sci.

[20] Valeur B, Berberan-Santos MRN (2011). A brief history of fluorescence and phosphorescence before the emergence of quantum theory. J Chem Educ.

[21] Blaaderen AV, Vrij A (1992). Synthesis and characterization of colloidal dispersions of fluorescent, monodisperse silica spheres. Langmuir..

[22] Burns A, Sengupta P, Zedayko T, Baird B, Wiesner U (2006). Core/Shell fluorescent silica nanoparticles for chemical sensing: towards single-particle laboratories. Small..

[23] Jiao L, Song F, Zhang B, Ning H, Cui J, Peng X (2017). Improving the brightness and photostability of NIR fluorescent silica nanoparticles through rational fine-tuning of the covalent encapsulation methods. J Mater Chem B.

[24] Geng J, Goh CC, Qin W, Liu R, Tomczak N, Ng LG (2015). Silica shelled and block copolymer encapsulated red-emissive AIE nanoparticles with 50% quantum yield for two-photon excited vascular imaging. Chem Commun (Camb).

[25] Bai Z, Chen R, Si P, Huang Y, Sun H, Kim DH (2013). Fluorescent pH sensor based on Ag@SiO2 core-shell nanoparticle. ACS Appl Mater Interfaces.

[26] Rajiv K, Indrajit R, Ohulchanskyy TY, Goswami LN, Bonoiu AC, Bergey EJ (2008). Covalently dye-linked, surface-controlled, and bioconjugated organically modified silica nanoparticles as targeted probes for optical imaging. ACS Nano.

[27] Xiqi Z, Xiaoyong Z, Shiqi W, Meiying L, Lei T, Yen W (2012). Surfactant modification of aggregation-induced emission material as biocompatible nanoparticles: facile preparation and cell imaging. Nanoscale..

[28] Atabaev TS, Hong HTV, Hwang YH, Kim HK (2017). Ratiometric pH sensor based on fluorescent core–shell nanoparticles. J Nanosci Nanotechnol.

[29] Ding Y, Zhu W, Xu Y, Qian X (2015). A small molecular fluorescent sensor functionalized silica microsphere for detection and removal of mercury, cadmium, and lead ions in aqueous solutions. Sensors Actuators B Chem.

[30] Francis B, Neuhaus B, Reddy M, Epple M, Janiak C (2017). Amine-functionalized silica nanoparticles incorporating covalently linked visible-light excitable Eu3+−complexes: synthesis, characterization and cell uptake studies. Eur J Inorg Chem.

[31] Li Y, Jiao J, Yan P, Liu L, Wang J, Wang Y (2018). Synthesis and tunable photoresponse for core-shell structured NaGdF 4 :Yb,Er@SiO 2 @Eu (TTA) 3 Phen nanocomplexes. Scr Mater.

[32] Shi M, Xia L, Chen Z, Lv F, Zhu H, Wei F (2017). Europium-doped mesoporous silica nanosphere as an immune-modulating osteogenesis/angiogenesis agent. Biomaterials..

[33] Salgueiriño-Maceira V, Correa-Duarte MA, Spasova M, Liz-Marzán LM, Farle M (2010). Composite silica spheres with magnetic and luminescent functionalities. Adv Funct Mater.

[34] Sun C, Zhang Y, Ruan C, Yin C, Wang X, Wang Y (2016). Efficient and stable white LEDs with silica-coated inorganic perovskite quantum dots. Adv Mater.

[35] Wang HC, Lin SY, Tang AC, Singh BP, Tong HC, Chen CY (2016). Mesoporous silica particles integrated with all-inorganic CsPbBr3 perovskite quantum-dot nanocomposites (MP-PQDs) with high stability and wide color gamut used for backlight display. Angew Chem Int Ed Engl.

[36] Wang J, Zhang F, Wang Y, Yang Y, Liu X (2018). Efficient resistance against solid-state quenching of carbon dots towards white light emitting diodes by physical embedding into silica. Carbon..

[37] Cho W, Lee HJ, Choi S, Kim Y, Oh M (2014). Highly effective heterogeneous chemosensors of luminescent silica@coordination polymer core-shell micro-structures for metal ion sensing. Sci Rep.

[38] Jin Y, Lohstreter S, Pierce DT, Parisien J, Wu M, Hall C (2008). Silica nanoparticles with continuously tunable sizes: synthesis and size effects on cellular contrast imaging. Chem Mater.

[39] Rieter WJ, Kim JS, Taylor KM, An H, Lin W, Tarrant T (2007). Hybrid silica nanoparticles for multimodal imaging. Angew Chem Int Ed Engl..

[40] Mirenda M, Levi V, Bossi ML, Bruno L, Bordoni AV, Regazzoni AE (2013). Temperature response of luminescent tris (bipyridine) ruthenium (II)-doped silica nanoparticles. J Colloid Interface Sci.

[41] Savchuk O, Carvajal JJ, Cascales C, Aguilo M, Diaz F (2016). Benefits of silica core-shell structures on the temperature sensing properties of Er,Yb:GdVO4 up-conversion nanoparticles. ACS Appl Mater Interfaces.

[42] Chen Y, Lei B, Zheng M, Zhang H, Zhuang J, Liu Y (2015). A dual-emitting core–shell carbon dot–silica–phosphor composite for white light emission. Nanoscale..

[43] Ma Y, Li Y, Ma S, Zhong X (2014). Highly bright water-soluble silica coated quantum dots with excellent stability. J Mater Chem B.

[44] Selvan ST, Patra PK, Ang CY, Ying JY (2007). Synthesis of silica-coated semiconductor and magnetic quantum dots and their use in the imaging of live cells. Angew Chem.

[45] Ji B, Giovanelli E, Habert B, Spinicelli P, Nasilowski M, Xu X (2015). Non-blinking quantum dot with a plasmonic nanoshell resonator. Nat Nanotechnol.

[46] Wang N, Koh S, Jeong BG, Lee D, Kim WD, Park K (2017). Highly luminescent silica-coated CdS/CdSe/CdS nanoparticles with strong chemical robustness and excellent thermal stability. Nanotechnology..

[47] Chen H, Zhen Z, Tang W, Todd T, Chuang YJ, Wang L (2013). Label-free luminescent mesoporous silica nanoparticles for imaging and drug delivery. Theranostics..

[48] Kee YD, Selvan ST, Seong LS, Papaefthymiou GC, Darshan K, Ying JY (2005). Silica-coated nanocomposites of magnetic nanoparticles and quantum dots. J Am Chem Soc.

[49] Chang K, Men X, Chen H, Liu Z, Yin S, Qin W (2015). Silica-encapsulated semiconductor polymer dots as stable phosphors for white light-emitting diodes. J Mater Chem C.

[50] Yang L, Wang L, Cui C, Lei J, Zhang J (2016). Stober strategy for synthesizing multifluorescent organosilica nanocrystals. Chem Commun (Camb)..

[51] Chandra Sourov, Beaune Grégory, Shirahata Naoto, Winnik Françoise M. (2017). A one-pot synthesis of water soluble highly fluorescent silica nanoparticles. Journal of Materials Chemistry B.

[52] Hun X, Zhang Z (2007). Preparation of a novel fluorescence nanosensor based on calcein-doped silica nanoparticles, and its application to the determination of calcium in blood serum. Microchim Acta.

[53] Ha SW, Camalier CE, Beck GR Jr, Lee JK (2009) New method to prepare very stable and biocompatible fluorescent silica nanoparticles. Chem Commun 2881–2883 10.1039/b902195g PMC2879016 19436897

[54] Hong Y, Lam JWY, Tang BZ (2011). Aggregation-induced emission. Chem Soc Rev.

[55] Inagaki S, Ohtani O, Goto Y, Okamoto K, Ikai M, Yamanaka K (2009). Light harvesting by a periodic mesoporous organosilica chromophore. Angew Chem Int Ed Engl..

[56] Huang CH (2010). Rare earth coordination chemistry.

[57] Baggaley E, Weinstein JA, Williams JAG (2012). Lighting the way to see inside the live cell with luminescent transition metal complexes. Coord Chem Rev.

[58] Kalyani N. Thejo, Swart Hendrik, Dhoble S.J. (2017). Future Prospects of Organic Light-Emitting Diodes. Principles and Applications of Organic Light Emitting Diodes (OLEDs).

[59] Sedlmeier A, Gorris HH (2015). Surface modification and characterization of photon-upconverting nanoparticles for bioanalytical applications. Chem Soc Rev.

[60] Ezquerro Cintia, Fresta Elisa, Serrano Elena, Lalinde Elena, García-Martínez Javier, Berenguer Jesús R., Costa Rubén D. (2019). White-emitting organometallo-silica nanoparticles for sun-like light-emitting diodes. Materials Horizons.

[61] Li T, Moon J, Morrone AA, Mecholsky JJ, Talham DR, Adair JH (1999). Preparation of Ag/SiO2 Nanosize composites by a reverse micelle and sol–gel technique. Langmuir..

[62] Boyer D, Tamarat P, Maali A, Lounis B, Orrit M (2002). Photothermal imaging of nanometer-sized metal particles among scatterers. Science..

[63] Stöber W, Fink A, Bohn E (1968). Controlled growth of monodisperse silica spheres in the micron size range. J Colloid Interface Sci.

[64] Liz-Marzá LM, Giersig M, Mulvaney P (1996). Synthesis of nanosized gold–silica core–shell particles. Langmuir..

[65] Leng Meiying, Yang Ying, Zeng Kai, Chen Zhengwu, Tan Zhifang, Li Shunran, Li Jinghui, Xu Bing, Li Dengbing, Hautzinger Matthew P., Fu Yongping, Zhai Tianyou, Xu Ling, Niu Guangda, Jin Song, Tang Jiang (2017). All-Inorganic Bismuth-Based Perovskite Quantum Dots with Bright Blue Photoluminescence and Excellent Stability. Advanced Functional Materials.

[66] Bagwe RP, Khilar KC (1997). Effects of the intermicellar exchange rate and cations on the size of silver chloride nanoparticles formed in reverse micelles of AOT. Langmuir..

[67] Osseo-Asare K, Arriagada FJ (1990). Preparation of SiO2 nanoparticles in a non-ionic reverse micellar system. Colloids Surfaces.

[68] Wang L, Zhang H, Zhou X, Liu Y, Lei B (2016). Preparation, characterization and oxygen sensing properties of luminescent carbon dots assembled mesoporous silica microspheres. J Colloid Interface Sci.

[69] Xie Bin, Zhang Jingjing, Chen Wei, Hao Junjie, Cheng Yanhua, Hu Run, Wu Dan, Wang Kai, Luo Xiaobing (2017). Realization of wide circadian variability by quantum dots-luminescent mesoporous silica-based white light-emitting diodes. Nanotechnology.

[70] Aldalbahi A, Rahaman M, Ansari AA (2019). Mesoporous silica modified luminescent Gd 2 O 3: Eu nanoparticles: physicochemical and luminescence properties. J Sol-Gel Sci Technol.

[71] Cao L, Jiang H, Song H, Li Z, Miao G (2010). Thermal CVD synthesis and photoluminescence of SiC/SiO2 core–shell structure nanoparticles. J Alloys Compd.

[72] Shahabi S, Treccani L, Rezwan K (2015). Amino acid-catalyzed seed regrowth synthesis of photostable high fluorescent silica nanoparticles with tunable sizes for intracellular studies. J Nanopart Res.

[73] Quan B, Lee C, Yoo JS, Piao Y (2017). Facile scalable synthesis of highly monodisperse small silica nanoparticles using alkaline buffer solution and its application for efficient sentinel lymph node mapping. J Mater Chem B.

[74] Dong WL, Yoo BR (2016). Advanced silica/polymer composites: materials and applications. J Ind Eng Chem.

[75] Wang J, Li Z, Zhang S, Yan S, Cao B, Wang Z (2017). Enhanced NH 3 gas-sensing performance of silica modified CeO2 nanostructure based sensors. Sensors Actuators B Chem.

[76] Liang J, Hu Y, Wu Y, Chen H (2014). Facile formation of superhydrophobic silica-based surface on aluminum substrate with tetraethylorthosilicate and vinyltriethoxysilane as co-precursor and its corrosion resistant performance in corrosive NaCl aqueous solution. Surf Coat Technol.

[77] Ma M, Zheng S, Chen H, Yao M, Zhang K, Jia X (2014). A combined “RAFT” and “graft from” polymerization strategy for surface modification of mesoporous silica nanoparticles: towards enhanced tumor accumulation and cancer therapy efficacy. J Mater Chem B.

[78] Park J-H, Gu L, von Maltzahn G, Ruoslahti E, Bhatia SN, Sailor MJ (2009). Biodegradable luminescent porous silicon nanoparticles for in vivo applications. Nat Mater.

[79] Mikael P, Kanyi P, Shirley S, Jianghong R (2015). Semiconducting polymer nanoparticles with persistent near-infrared luminescence for in vivo optical imaging. Angew Chem.

[80] Kim IY, Joachim E, Choi H, Kim K (2015). Toxicity of silica nanoparticles depends on size, dose, and cell type. Nanomedicine Nanotechnol Biol Med.

[81] Michael PK (2016) Silica, silicosis, and autoimmunity. Front Immunol 7:97. 10.3389/fimmu.2016.00097 PMC4786551 27014276

[82] Laranjeira M, Shirosaki Y, Yoshimatsu YS, Miyazaki T, Monteiro FJ (2017). Enhanced biosafety of silica coated gadolinium based nanoparticles. J Mater Sci Mater Med.

[83] Atabaev TS, Lee JH, Han DW, Choo KS, Jeon UB, Hwang JY (2016). Multicolor nanoprobes based on silica-coated gadolinium oxide nanoparticles with highly reduced toxicity. RSC Adv.

[84] Wells JD, Koopal LK, Keizer AD (2000). Monodisperse, nonporous, spherical silica particles. Colloids Surf A Physicochem Eng Aspects.

[85] Eunjoo J, Shinae J, Hyosook J, Jungeun L, Byungki K, Younghwan K (2010). White-light-emitting diodes with quantum dot color converters for display backlights. Adv Mater.

[86] Jiang K, Sun S, Zhang L, Lu Y, Wu A, Cai C (2015). Red, green, and blue luminescence by carbon dots: full-color emission tuning and multicolor cellular imaging. Angew Chem Int Ed Engl..

[87] Peng J, Li J, Xu W, Wang L, Su D, Teoh CL (2018). Silica nanoparticle-enhanced fluorescent sensor array for heavy metal ions detection in colloid solution. Anal Chem.

